# Supplementary figures and images for: On partial randomized response model using ranked set sampling
Source: PLoS One. 2022 Nov 29;17(11):e0277497. doi: 10.1371/journal.pone.0277497 (PMC9707803; doi:10.1371/journal.pone.0277497)

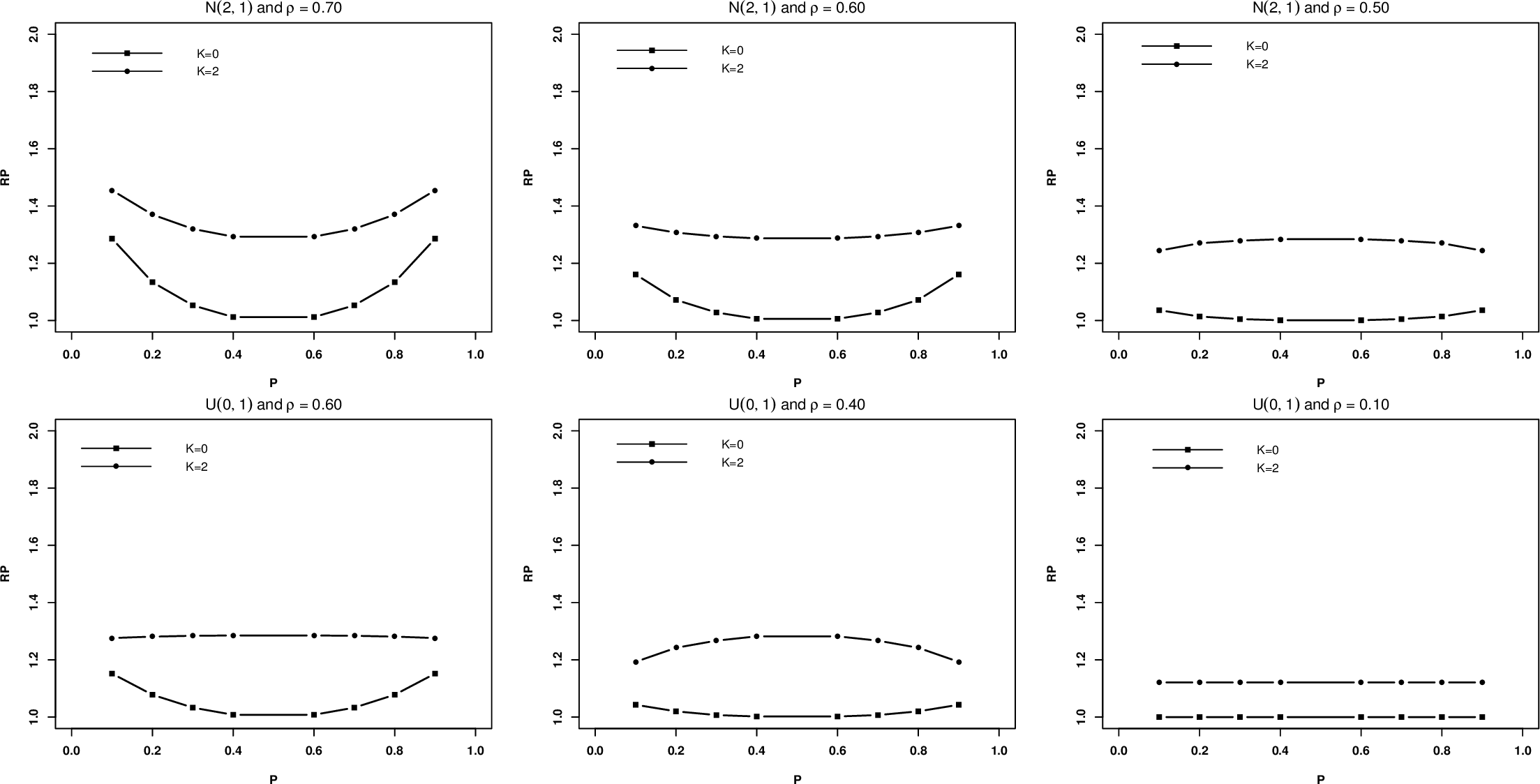

Supplement: S1 Fig — (TIF) [file pone.0277497.s001.tif]

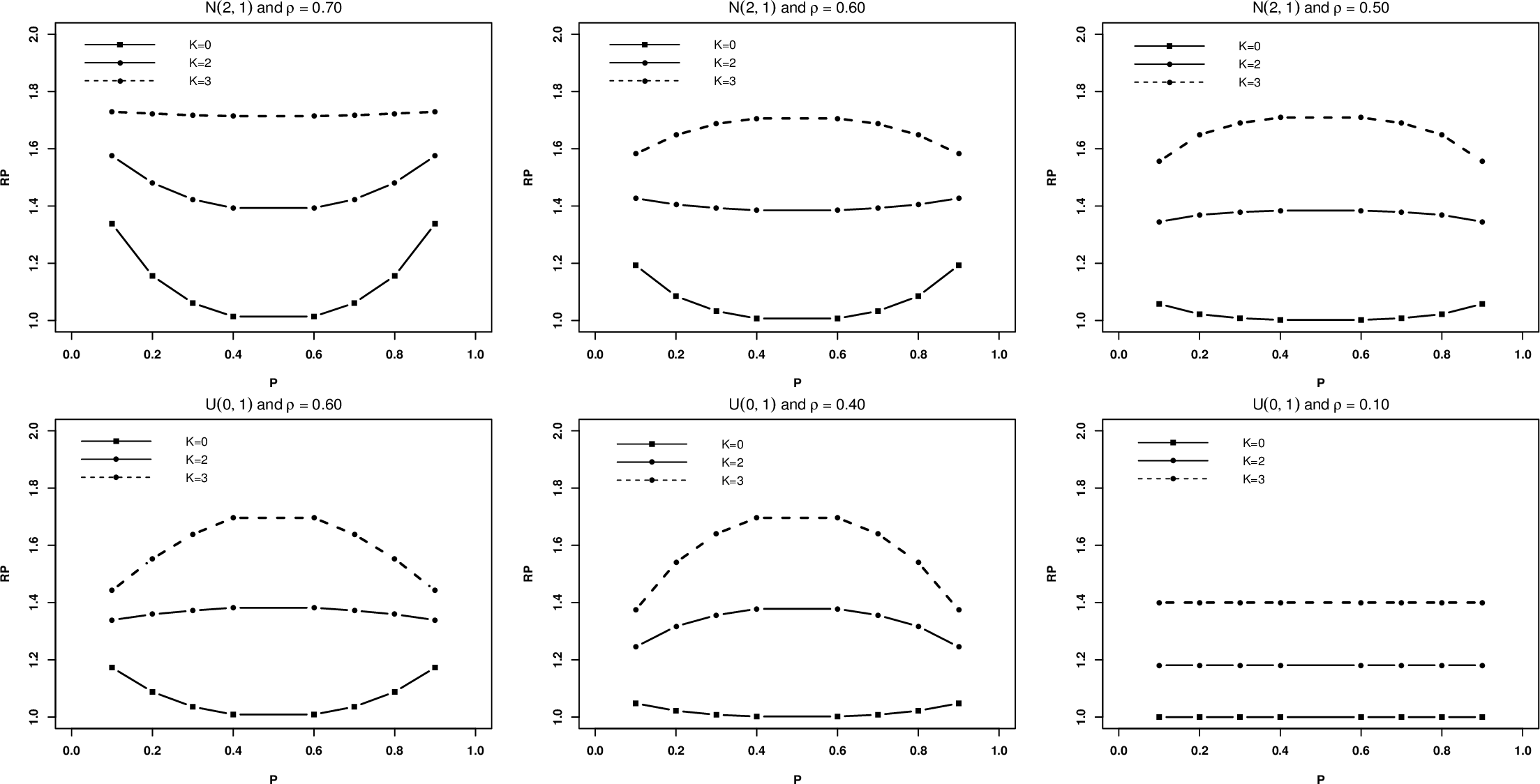

Supplement: S2 Fig — (TIF) [file pone.0277497.s002.tif]

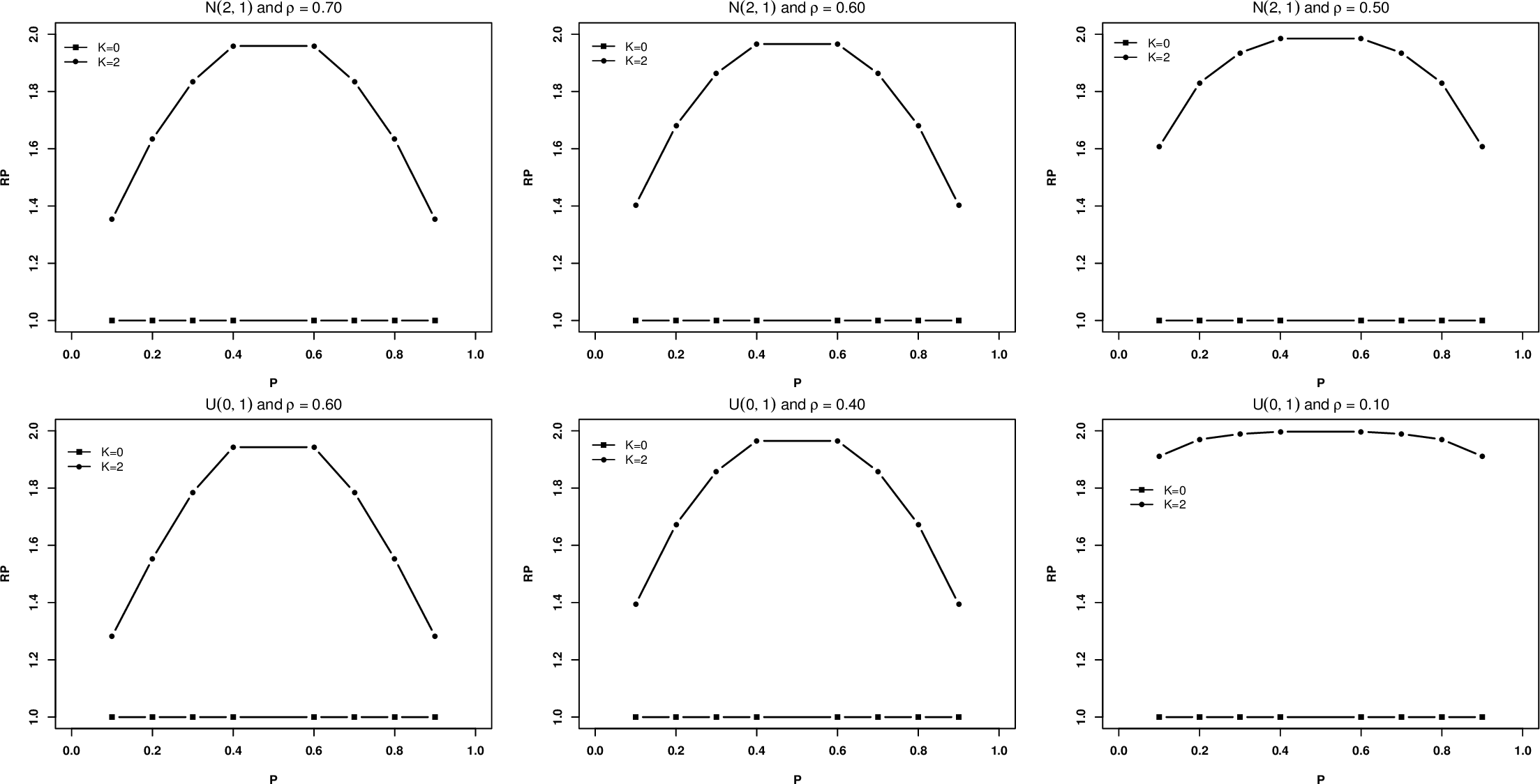

Supplement: S3 Fig — (TIF) [file pone.0277497.s003.tif]

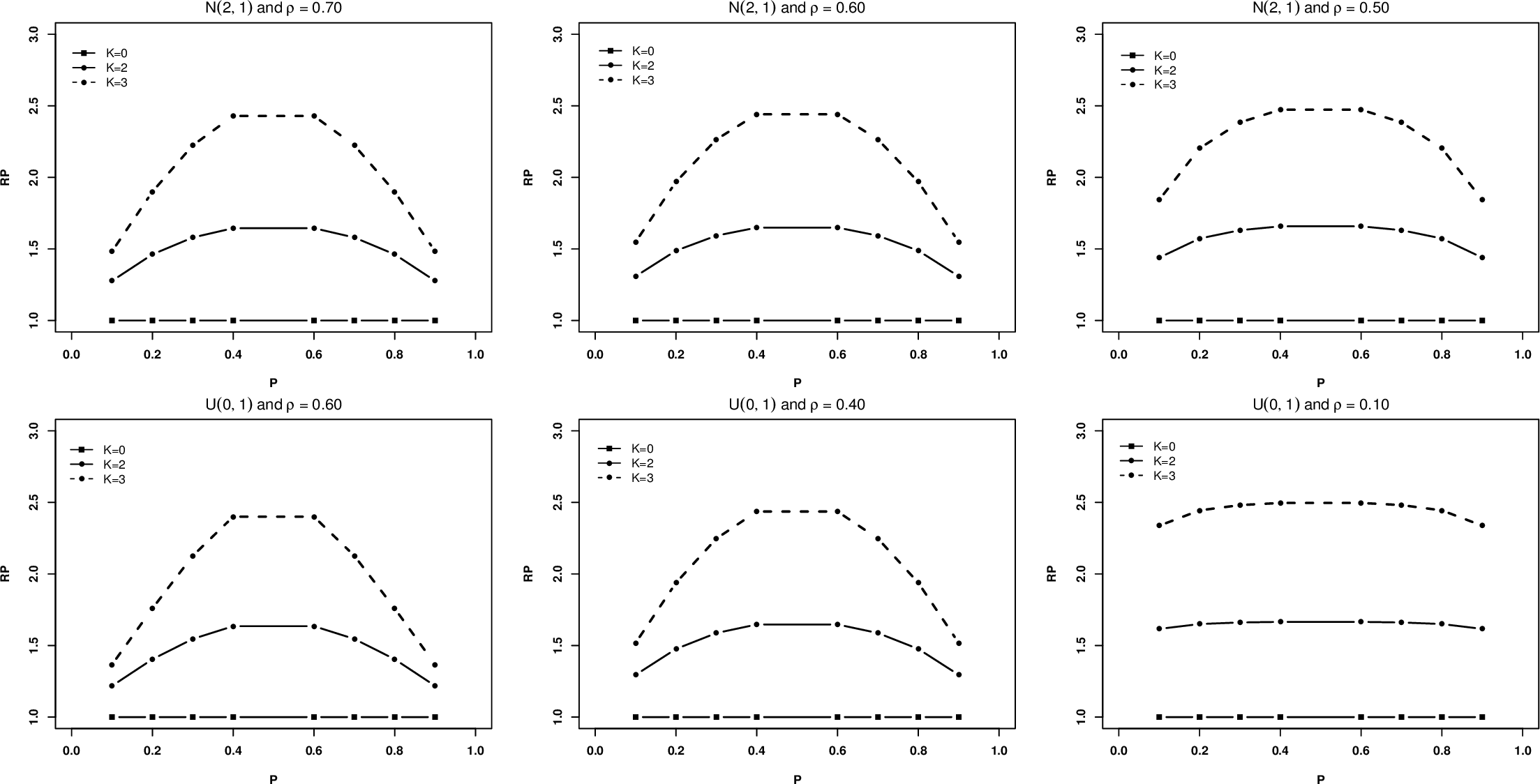

Supplement: S4 Fig — (TIF) [file pone.0277497.s004.tif]

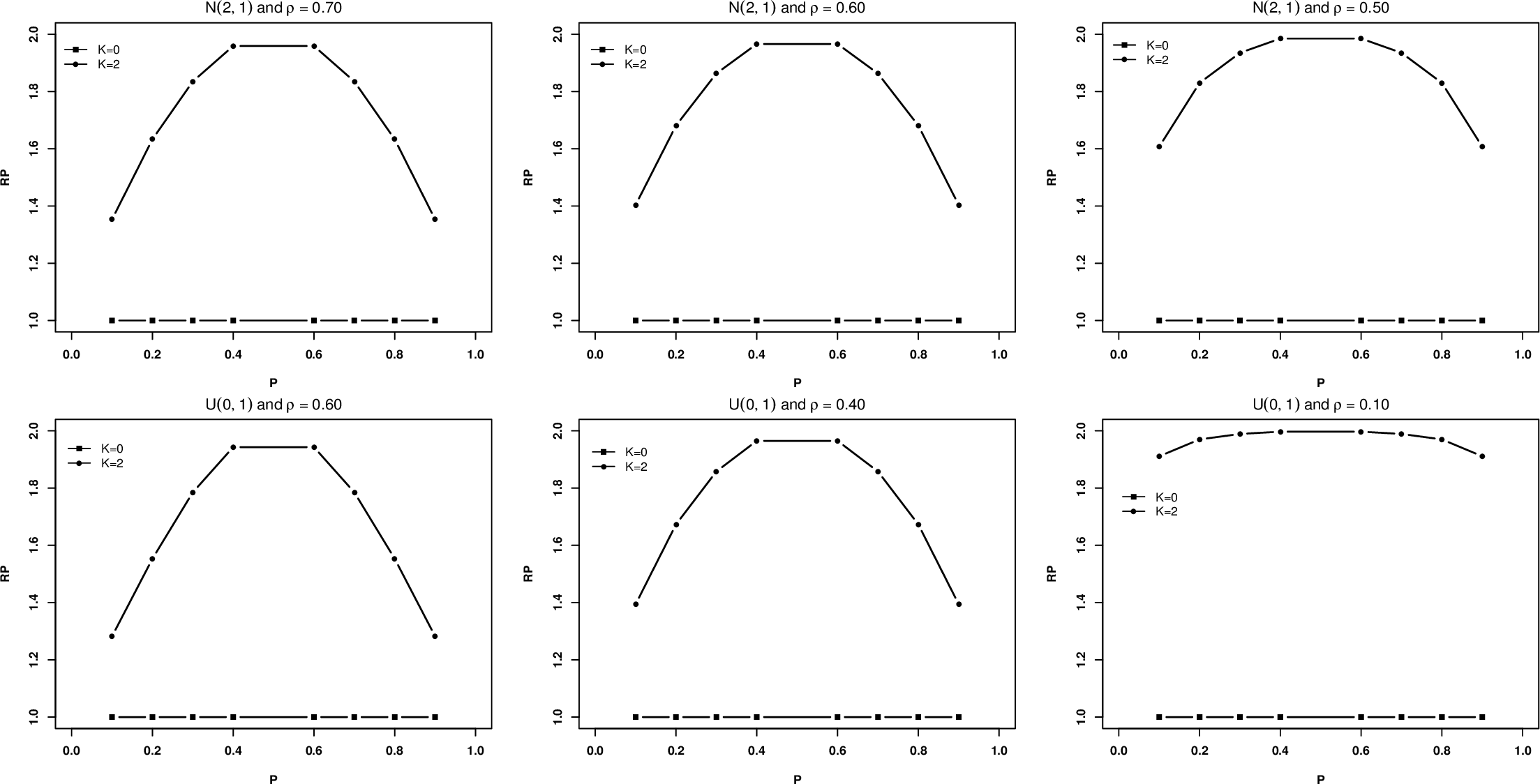

Supplement: S5 Fig — (TIF) [file pone.0277497.s005.tif]

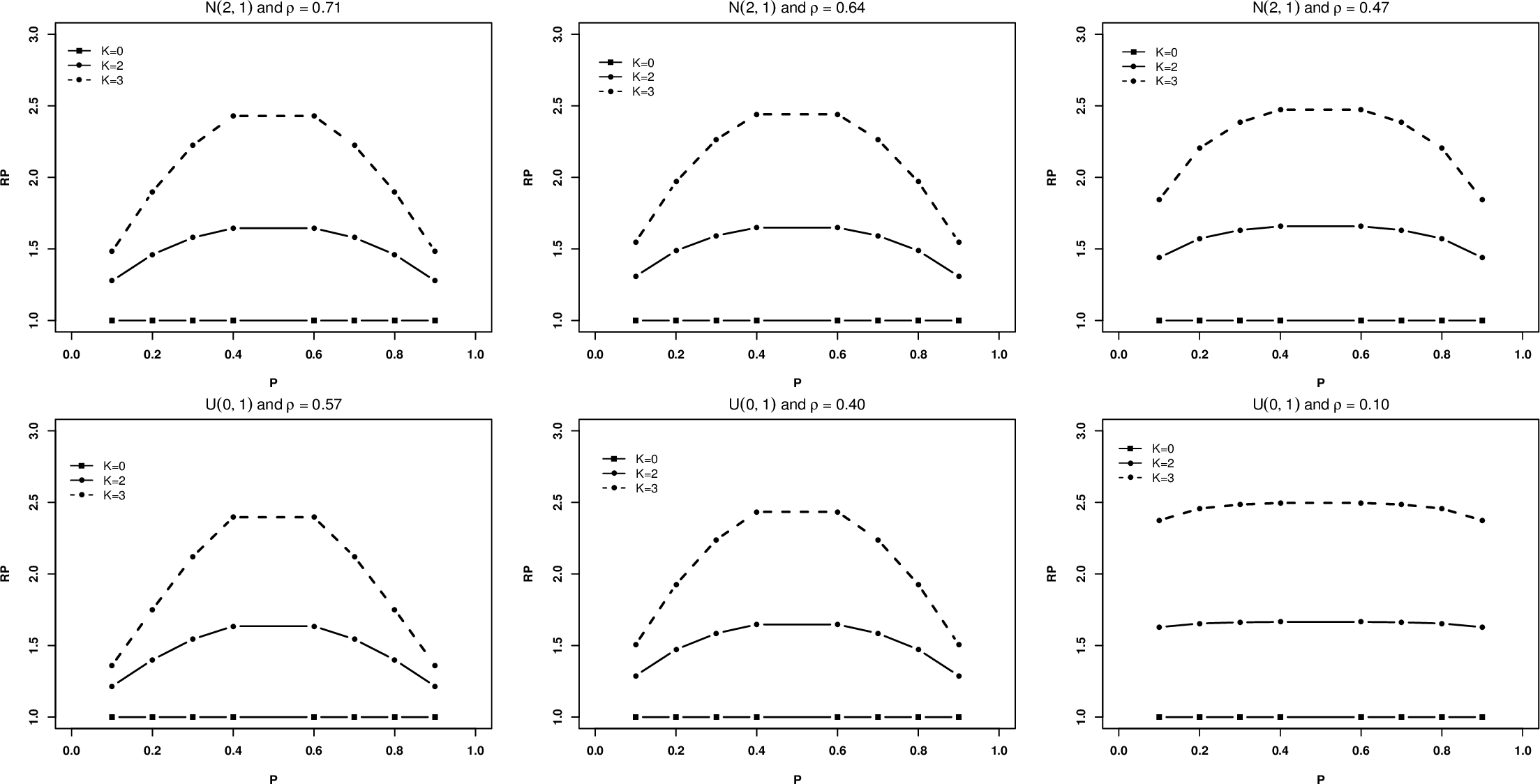

Supplement: S6 Fig — (TIF) [file pone.0277497.s006.tif]

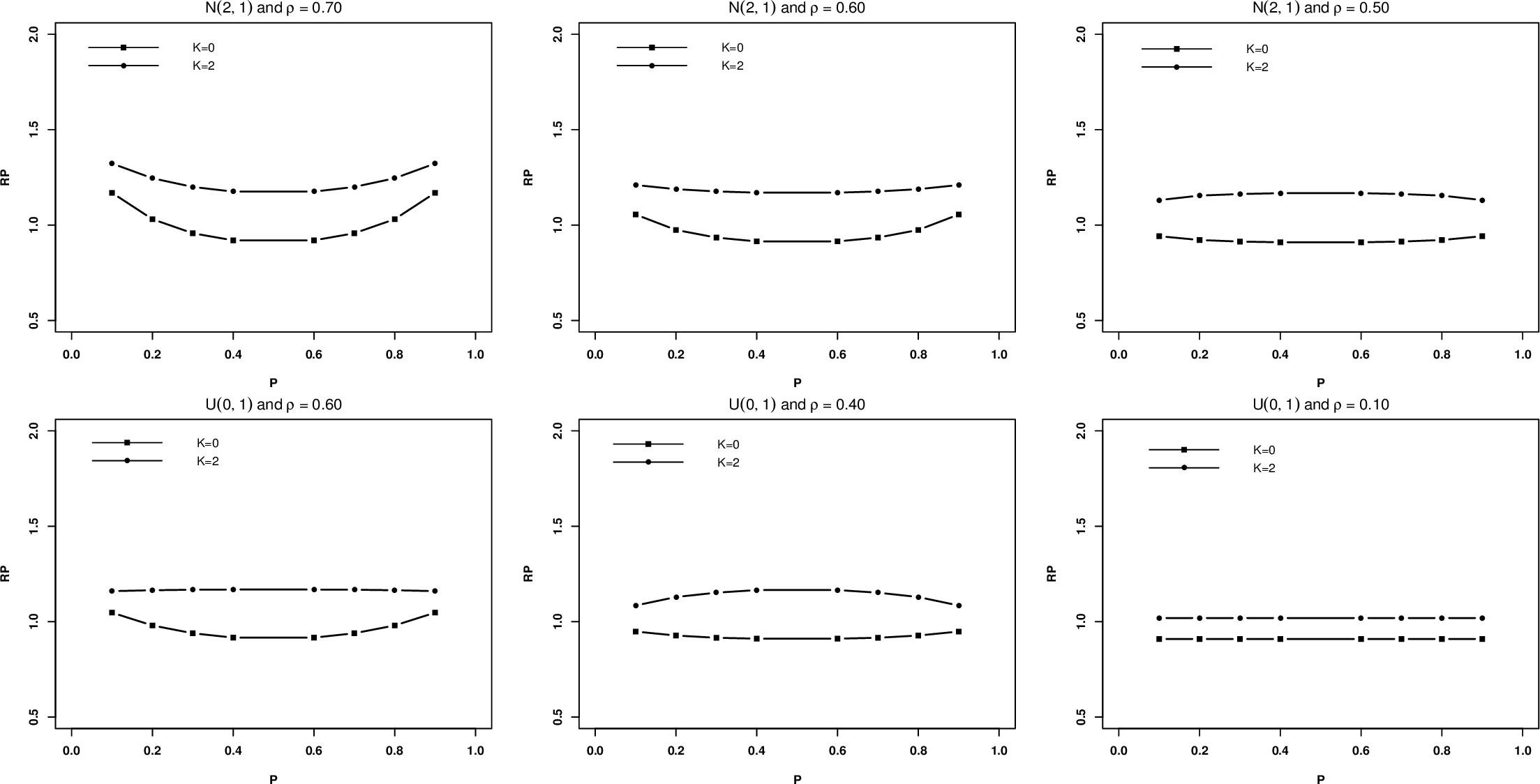

Supplement: S7 Fig — (TIF) [file pone.0277497.s007.tif]

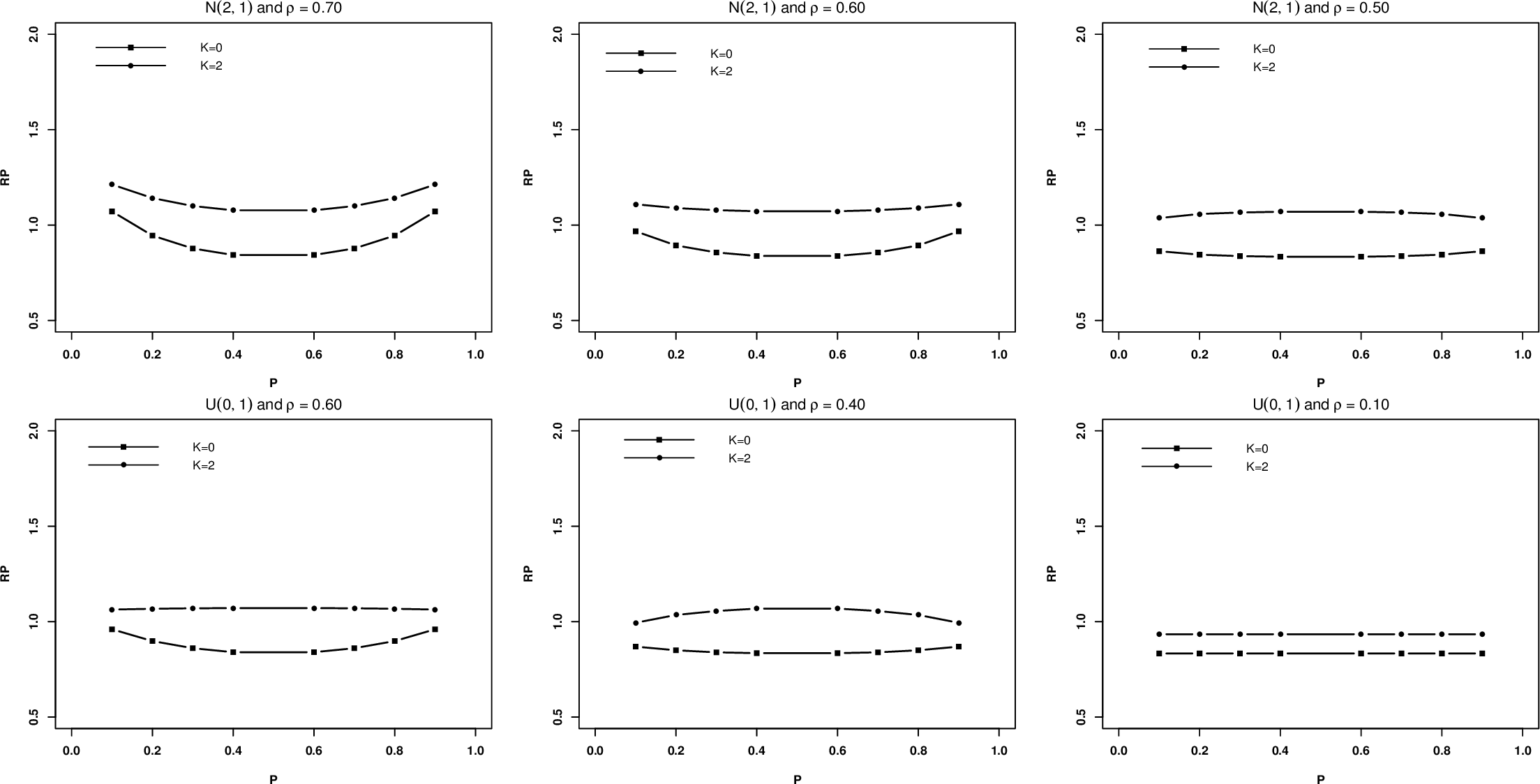

Supplement: S8 Fig — (TIF) [file pone.0277497.s008.tif]

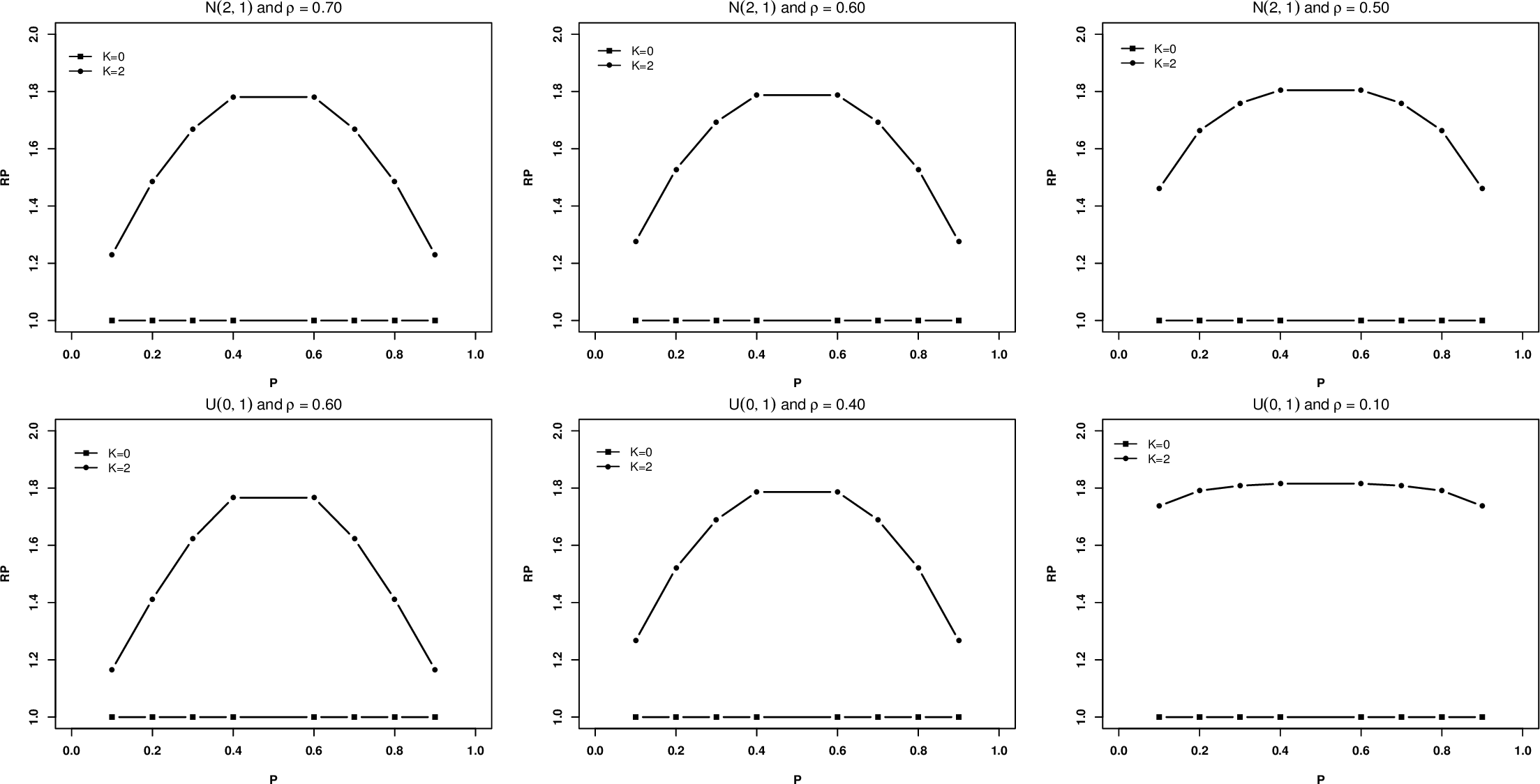

Supplement: S9 Fig — (TIF) [file pone.0277497.s009.tif]

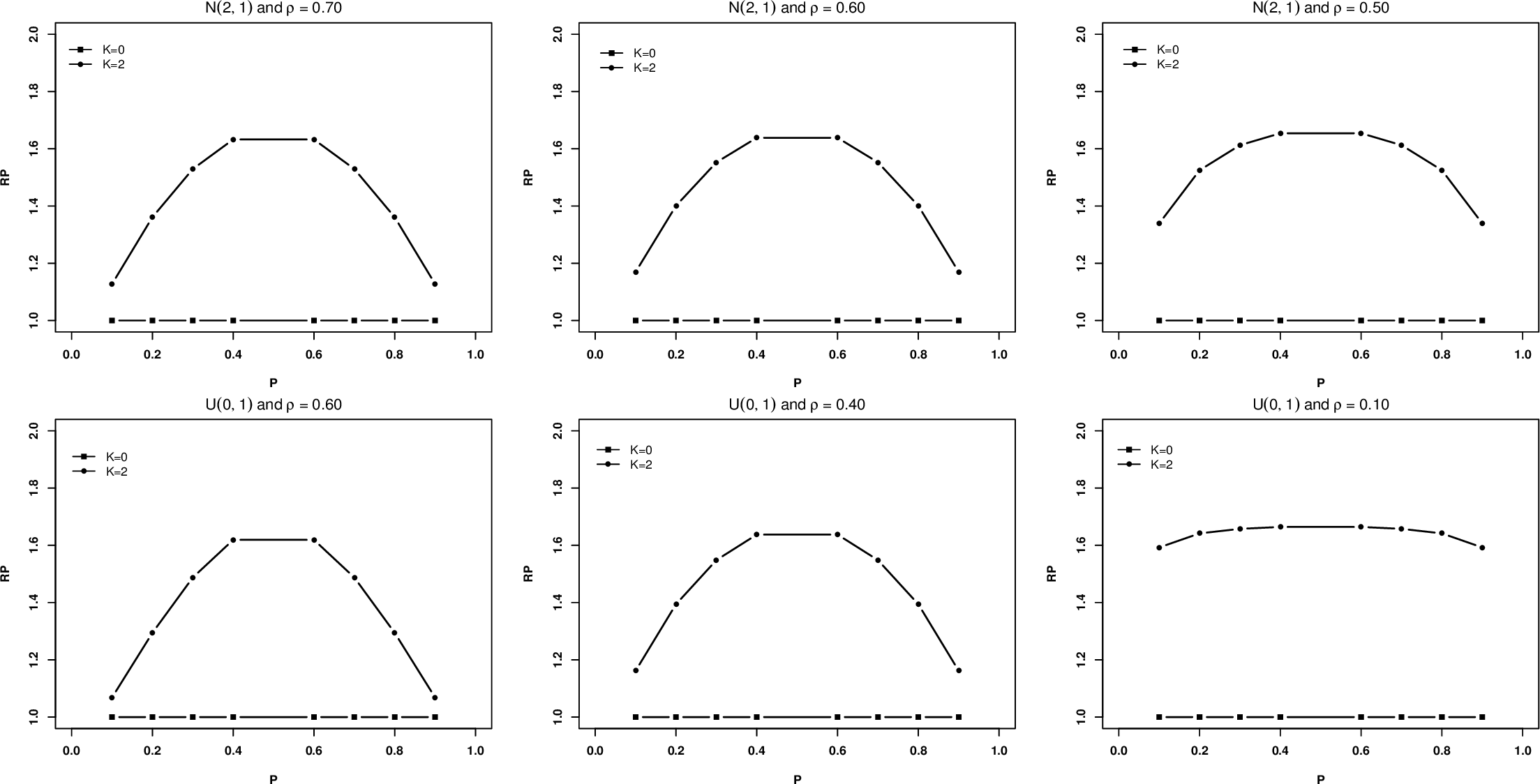

Supplement: S10 Fig — (TIF) [file pone.0277497.s010.tif]

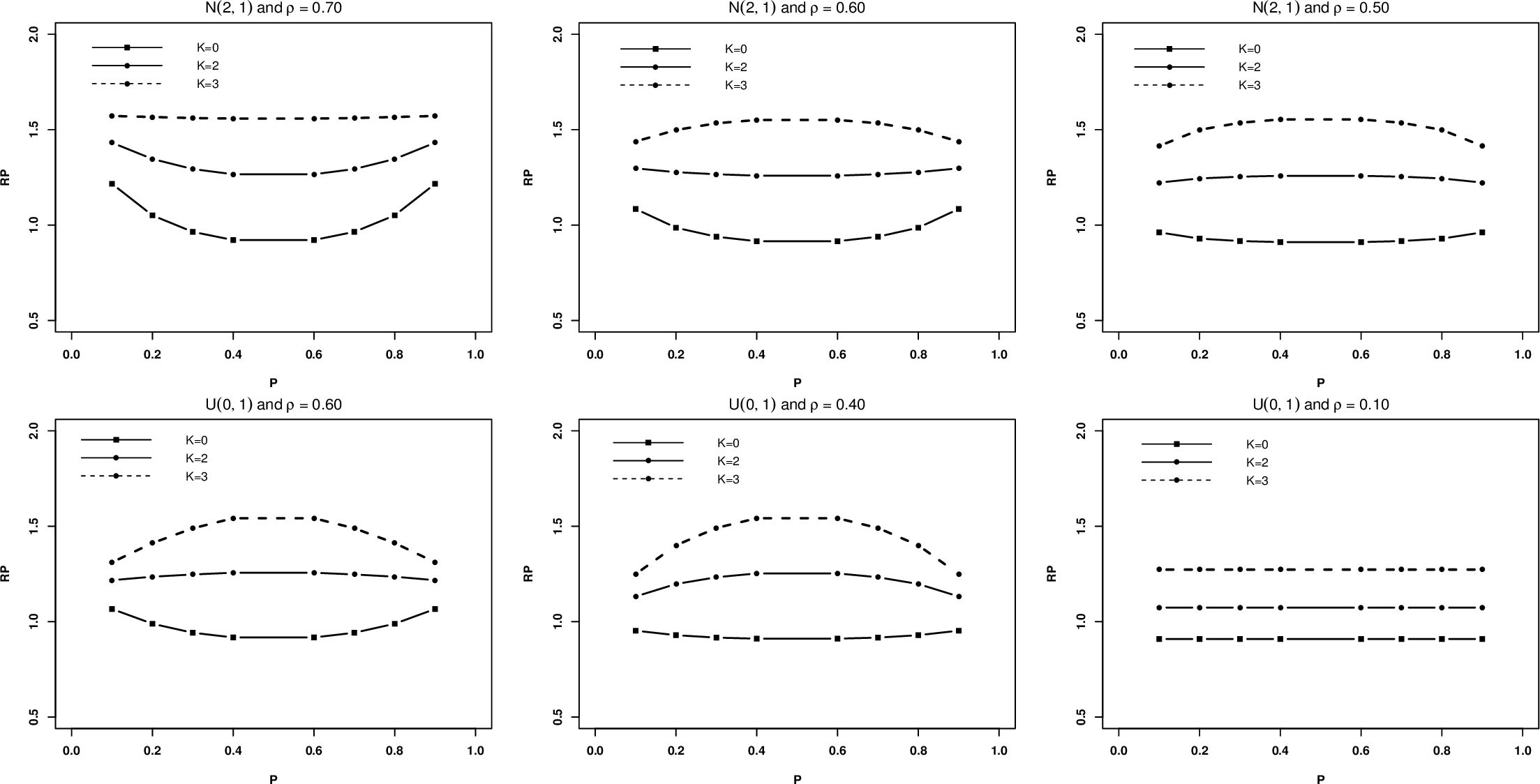

Supplement: S11 Fig — (TIF) [file pone.0277497.s011.tif]

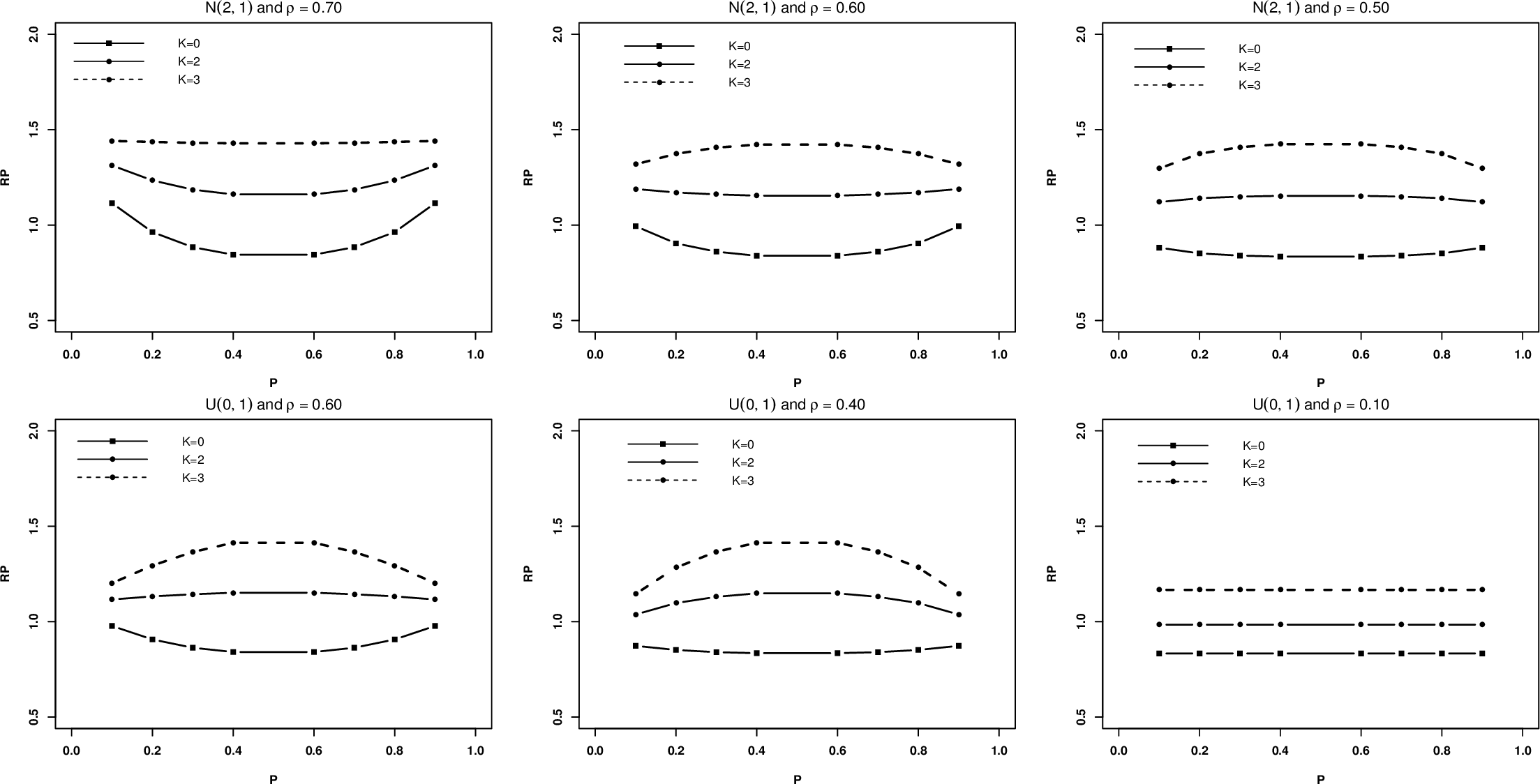

Supplement: S12 Fig — (TIF) [file pone.0277497.s012.tif]

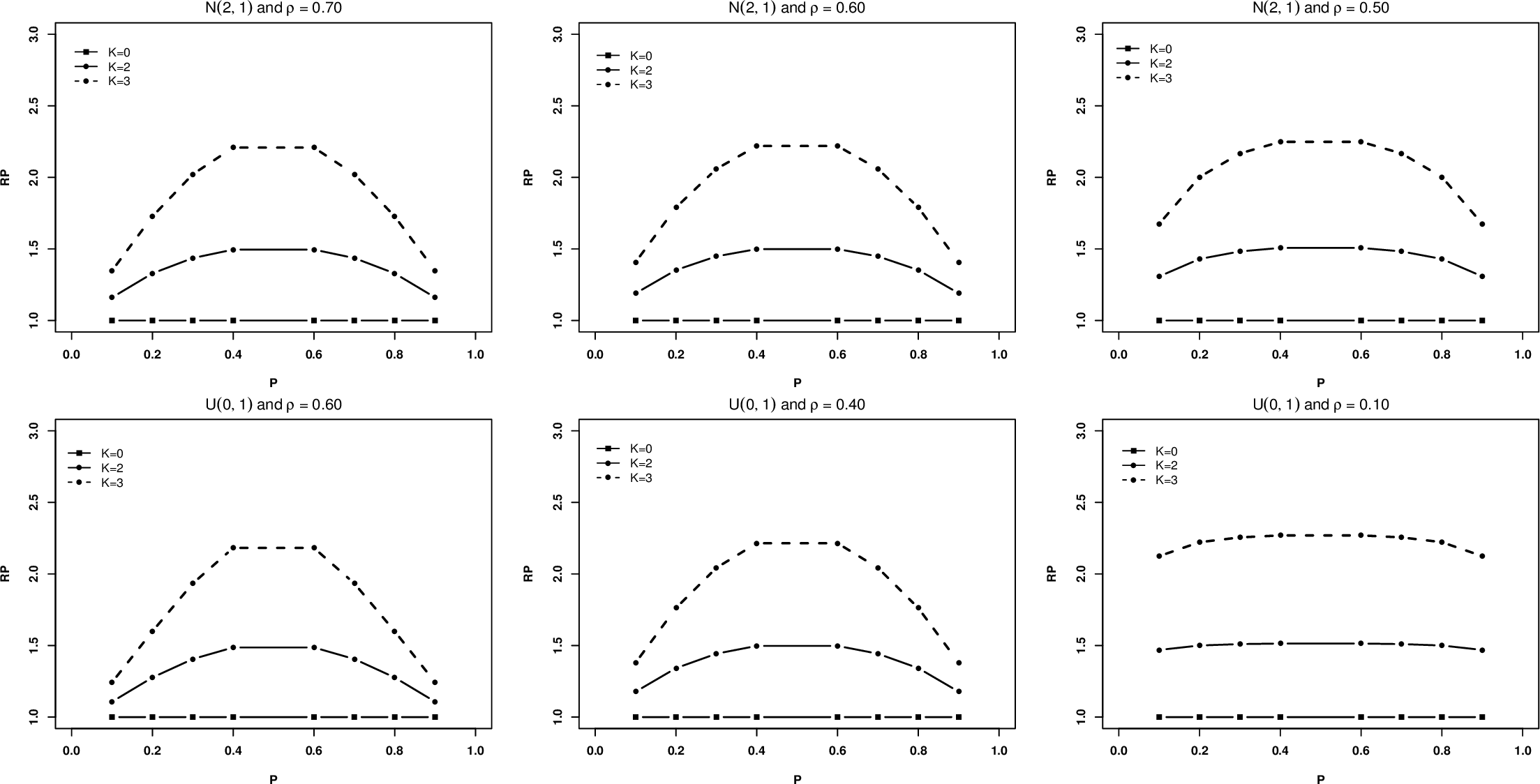

Supplement: S13 Fig — (TIF) [file pone.0277497.s013.tif]

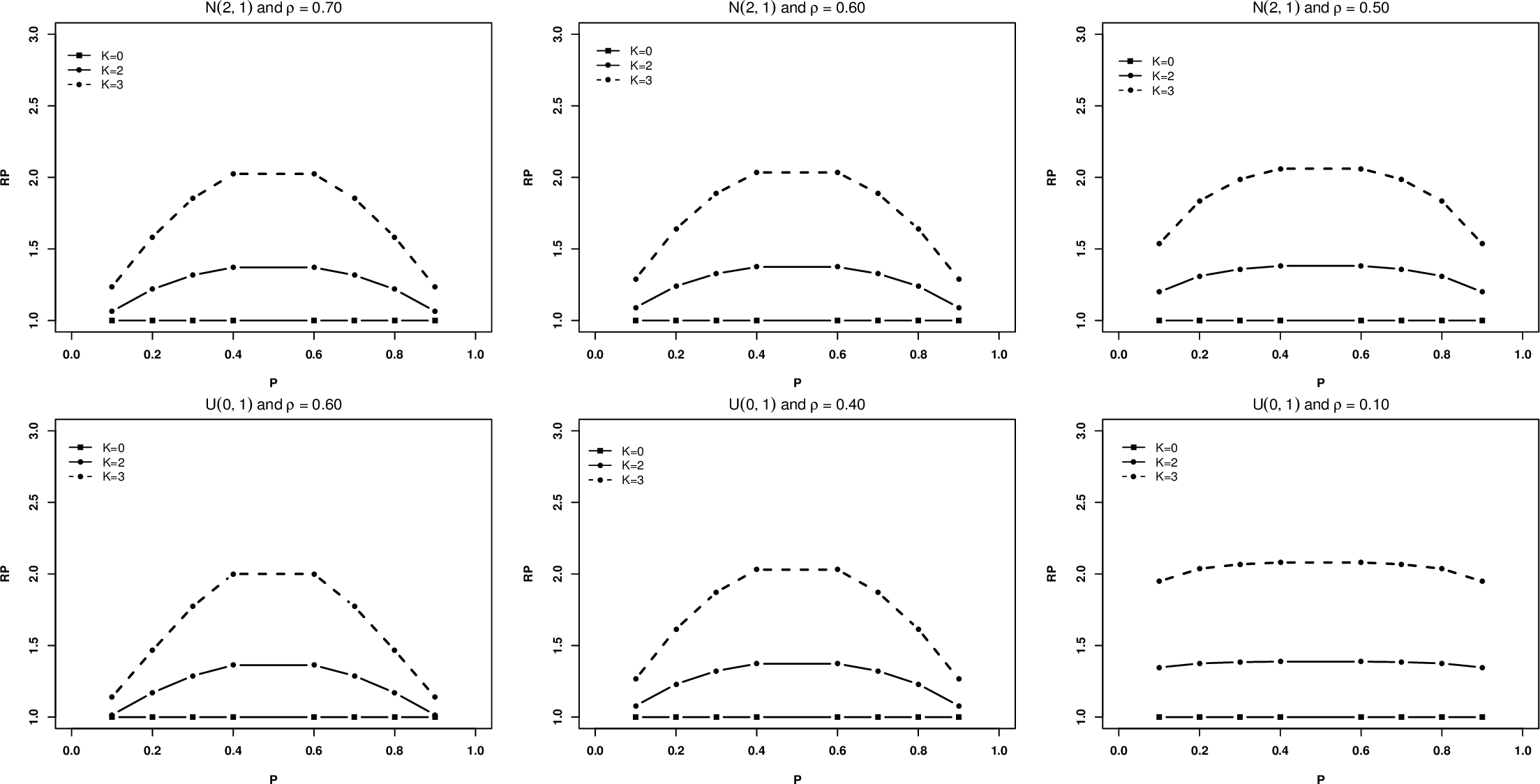

Supplement: S14 Fig — (TIF) [file pone.0277497.s014.tif]
