# Supplementary material for: On partial randomized response model using ranked set sampling
Source: PLoS One. 2022 Nov 29;17(11):e0277497. doi: 10.1371/journal.pone.0277497 (PMC9707803; doi:10.1371/journal.pone.0277497)
Supplement: S1 Table — (PDF) [file pone.0277497.s015.pdf]

Table S1 . A partial randomized response real data when $m = 4$, $k = 0$

r	m	Set Units				Obtained data
1	1	$\boxed{(Y^*_{[1]11}, 22)}$	$(Y_{[2]11}, 30)$	$(Y_{[3]11}, 32)$	$(Y_{[4]11}, 44)$	$Y^*_{[1]1} = 1$
	2	$(Y_{[1]21}, 19)$	$\boxed{(Y^*_{[2]21}, 29)}$	$(Y_{[3]21}, 30)$	$(Y_{[4]21}, 31)$	$Y^*_{[2]1} = 1$
	3	$(Y_{[1]31}, 22)$	$(Y_{[2]31}, 37)$	$\boxed{(Y^*_{[3]31}, 43)}$	$(Y_{[4]31}, 56)$	$Y^*_{[3]1} = 0$
	4	$(Y_{[1]41}, 29)$	$(Y_{[2]41}, 43)$	$(Y_{[3]41}, 51)$	$\boxed{(Y^*_{[4]41}, 51)}$	$Y^*_{[4]1} = 1$
2	1	$\boxed{(Y^*_{[1]12}, 33)}$	$(Y_{[2]12}, 35)$	$(Y_{[3]12}, 44)$	$(Y_{[4]12}, 49)$	$Y^*_{[1]2} = 0$
	2	$(Y_{[1]22}, 19)$	$\boxed{(Y^*_{[2]22}, 32)}$	$(Y_{[3]22}, 32)$	$(Y_{[4]22}, 47)$	$Y^*_{[2]2} = 0$
	3	$(Y_{[1]32}, 33)$	$(Y_{[2]32}, 47)$	$\boxed{(Y^*_{[3]22}, 41)}$	$(Y_{[4]32}, 60)$	$Y^*_{[3]2} = 1$
	4	$(Y_{[1]42}, 31)$	$(Y_{[2]42}, 35)$	$(Y_{[3]42}, 35)$	$\boxed{(Y^*_{[4]42}, 49)}$	$Y^*_{[4]2} = 1$
