# Supplementary material for: On partial randomized response model using ranked set sampling
Source: PLoS One. 2022 Nov 29;17(11):e0277497. doi: 10.1371/journal.pone.0277497 (PMC9707803; doi:10.1371/journal.pone.0277497)
Supplement: S2 Table — (PDF) [file pone.0277497.s016.pdf]

Table S2 . A partial randomized response real data when $m = 4$, $k = 2$

r	m	Set Units ($k = 2$)		Set Units ($m - k = 2$)		Obtained data
1	1	$\boxed{(Y_{[1]11}, 29)}$	$(Y_{[2]11}, 56)$	$\boxed{(Y^*_{[1]11}, 19)}$	$(Y_{[2]11}, 37)$	$Y_{[1]1} = 0$,$Y^*_{[1]1} = 0$
	2	$(Y_{[1]21}, 29)$	$\boxed{(Y_{[2]21}, 47)}$	$(Y_{[1]21}, 29)$	$\boxed{(Y^*_{[2]21}, 47)}$	$Y_{[2]1} = 1$,$Y^*_{[2]1} = 0$
2	1	$\boxed{(Y_{[1]12}, 19)}$	$(Y_{[2]12}, 35)$	$\boxed{(Y^*_{[1]12}, 40)}$	$(Y_{[2]12}, 60)$	$Y_{[1]2} = 0$,$Y^*_{[1]2} = 1$
	2	$(Y_{[1]22}, 23)$	$\boxed{(Y_{[2]22}, 43)}$	$(Y_{[1]22}, 37)$	$\boxed{(Y^*_{[2]22}, 47)}$	$Y_{[2]2} = 0$,$Y^*_{[2]2} = 1$
