# Supplementary material for: On partial randomized response model using ranked set sampling
Source: PLoS One. 2022 Nov 29;17(11):e0277497. doi: 10.1371/journal.pone.0277497 (PMC9707803; doi:10.1371/journal.pone.0277497)
Supplement: S3 Table — (PDF) [file pone.0277497.s017.pdf]

Table S3 . RP of $\hat{\pi}_k$ w.r.t $\hat{\pi}_w$ and $\hat{\pi}_a$ from AIDS data set

	RP($\hat{\pi}_k, \hat{\pi}_w$)		RP($\hat{\pi}_k, \hat{\pi}_a$)	
	$m = 4$	$m = 5$	$m = 4$	$m = 5$
$k = 0$	1.098	1.152	1.000	1.000
$k = 2$	1.693	1.465	1.542	1.381
$k = 3$	–	1.691	–	1.706
