# Supplementary material for: On partial randomized response model using ranked set sampling
Source: PLoS One. 2022 Nov 29;17(11):e0277497. doi: 10.1371/journal.pone.0277497 (PMC9707803; doi:10.1371/journal.pone.0277497)
Supplement: Attachment — Submitted filename: PONE-D-22-10865_reviewer (1).pdf [file pone.0277497.s018.pdf]

# PLOS ONE

## On partial randomized response model using ranked set sampling

--Manuscript Draft--

| | |
|---|---|
| **Manuscript Number:** | PONE-D-22-10865 |
| **Article Type:** | Research Article |
| **Full Title:** | On partial randomized response model using ranked set sampling |
| **Short Title:** | On partial randomized response model using ranked set sampling |
| **Corresponding Author:** | Azhar Mehmood Abbasi<br>Quaid-i-Azam University<br>Islamabad, PAKISTAN |
| **Keywords:** | |
| **Abstract:** | In this paper, we propose a partial randomized response technique to collect reliable sensitive data for estimation of population proportion in ranked set sampling (RSS) scheme using auxiliary information. The idea is to increase confidence and (or) co-operation of the respondents by providing them the option of both `direct' and `randomized' response for the inquired sensitive question. This option is quite logical because perception of sensitive (insensitive) inquiry can vary among respondents. Then properties of the proposed method are discussed and compared with existing randomized response technique suggested by Warner1965. Cost analysis is also carried out to prove supremacy of the suggested method. Finally, an application to clinical trial on AIDS is included. |
| **Order of Authors:** | Azhar Mehmood Abbasi |
| | Yousuf Shad |
| **Additional Information:** | |
| **Question** | **Response** |
| **Financial Disclosure**<br><br>Enter a financial disclosure statement that describes the sources of funding for the work included in this submission. Review the submission guidelines for detailed requirements. View published research articles from *PLOS ONE* for specific examples.<br><br>This statement is required for submission and **will appear in the published article** if the submission is accepted. Please make sure it is accurate. | The authors received no specific funding for this work |

Powered by Editorial Manager® and ProduXion Manager® from Aries Systems Corporation

| | |
|---|---|
| **Unfunded studies**<br>Enter: *The author(s) received no specific funding for this work.*<br><br>**Funded studies**<br>Enter a statement with the following details:<br>• Initials of the authors who received each award<br>• Grant numbers awarded to each author<br>• The full name of each funder<br>• URL of each funder website<br>• Did the sponsors or funders play any role in the study design, data collection and analysis, decision to publish, or preparation of the manuscript?<br>• **NO** - Include this sentence at the end of your statement: *The funders had no role in study design, data collection and analysis, decision to publish, or preparation of the manuscript.*<br>• **YES** - Specify the role(s) played.<br><br>* typeset | |
| **Competing Interests**<br><br>Use the instructions below to enter a competing interest statement for this submission. On behalf of all authors, disclose any competing interests that could be perceived to bias this work—acknowledging all financial support and any other relevant financial or non-financial competing interests.<br><br>This statement is required for submission and **will appear in the published article** if the submission is accepted. Please make sure it is accurate and that any funding sources listed in your Funding Information later in the submission form are also declared in your Financial Disclosure statement.<br><br>View published research articles from *PLOS ONE* for specific examples. | The authors have declared that no competing interests exist. |

| | |
|---|---|
| **NO authors have competing interests**<br><br>Enter: *The authors have declared that no competing interests exist.*<br><br>**Authors with competing interests**<br><br>Enter competing interest details beginning with this statement:<br><br>*I have read the journal's policy and the authors of this manuscript have the following competing interests: [insert competing interests here]*<br><br>* typeset | |
| **Ethics Statement**<br><br>Enter an ethics statement for this submission. This statement is required if the study involved:<br><br>• Human participants<br>• Human specimens or tissue<br>• Vertebrate animals or cephalopods<br>• Vertebrate embryos or tissues<br>• Field research<br><br>Write "N/A" if the submission does not require an ethics statement.<br><br>General guidance is provided below. Consult the submission guidelines for detailed instructions. **Make sure that all information entered here is included in the Methods section of the manuscript.** | NA |

Powered by Editorial Manager® and ProduXion Manager® from Aries Systems Corporation

| | |
|---|---|
| **Format for specific study types**<br><br>**Human Subject Research (involving human participants and/or tissue)**<br>• Give the name of the institutional review board or ethics committee that approved the study<br>• Include the approval number and/or a statement indicating approval of this research<br>• Indicate the form of consent obtained (written/oral) or the reason that consent was not obtained (e.g. the data were analyzed anonymously)<br><br>**Animal Research (involving vertebrate animals, embryos or tissues)**<br>• Provide the name of the Institutional Animal Care and Use Committee (IACUC) or other relevant ethics board that reviewed the study protocol, and indicate whether they approved this research or granted a formal waiver of ethical approval<br>• Include an approval number if one was obtained<br>• If the study involved *non-human primates*, add *additional details* about animal welfare and steps taken to ameliorate suffering<br>• If anesthesia, euthanasia, or any kind of animal sacrifice is part of the study, include briefly which substances and/or methods were applied<br><br>**Field Research**<br><br>Include the following details if this study involves the collection of plant, animal, or other materials from a natural setting:<br>• Field permit number<br>• Name of the institution or relevant body that granted permission | |
| **Data Availability**<br><br>Authors are required to make all data underlying the findings described fully available, without restriction, and from the time of publication. PLOS allows rare exceptions to address legal and ethical concerns. See the PLOS Data Policy and FAQ for detailed information. | Yes - all data are fully available without restriction |

| | |
|---|---|
| A Data Availability Statement describing where the data can be found is required at submission. Your answers to this question constitute the Data Availability Statement and **will be published in the article**, if accepted.<br><br>**Important:** Stating 'data available on request from the author' is not sufficient. If your data are only available upon request, select 'No' for the first question and explain your exceptional situation in the text box.<br><br>Do the authors confirm that all data underlying the findings described in their manuscript are fully available without restriction? | |
| **Describe where the data may be found in full sentences. If you are copying our sample text, replace any instances of XXX with the appropriate details.**<br><br>• If the data are **held or will be held in a public repository**, include URLs, accession numbers or DOIs. If this information will only be available after acceptance, indicate this by ticking the box below. For example: *All XXX files are available from the XXX database (accession number(s) XXX, XXX.*).<br>• If the data are all contained **within the manuscript and/or Supporting Information files**, enter the following: *All relevant data are within the manuscript and its Supporting Information files.*<br>• If neither of these applies but you are able to provide **details of access elsewhere**, with or without limitations, please do so. For example:<br><br>*Data cannot be shared publicly because of [XXX]. Data are available from the XXX Institutional Data Access / Ethics Committee (contact via XXX) for researchers who meet the criteria for access to confidential data.*<br><br>*The data underlying the results presented in the study are available from (include the name of the third party* | Manuscript |

| | |
|---|---|
| *and contact information or URL).*<br>• This text is appropriate if the data are owned by a third party and authors do not have permission to share the data.<br><br>* typeset | |
| Additional data availability information: | Tick here if the URLs/accession numbers/DOIs will be available only after acceptance of the manuscript for publication so that we can ensure their inclusion before publication. |

Powered by Editorial Manager® and ProduXion Manager® from Aries Systems Corporation

Manuscript Click here to access/download;Manuscript;Manuscript.pdf

# On partial randomized response model using ranked set sampling

Azhar Mehmood Abbasi*† and Muhammad Yousaf Shad†

†Department of Statistics, Quaid-i-Azam University, Islamabad, Pakistan.

**Abstract**

In this paper, we propose a partial randomized response technique to collect reliable sensitive data for estimation of population proportion in ranked set sampling (RSS) scheme using auxiliary information. The idea is to increase confidence and (or) co-operation of the respondents by providing them the option of both 'direct' and 'randomized' response for the inquired sensitive question. This option is quite logical because perception of sensitive (insensitive) inquiry can vary among respondents. Then properties of the proposed method are discussed and compared with existing randomized response technique suggested by Warner (1965). Cost analysis is also carried out to prove supremacy of the suggested method. Finally, an application to clinical trial on AIDS is included.

**Keywords**: simple random sampling; ranked set sampling; link function; relative precision; relative efficiency; auxiliary variable; direct response; randomized response.

# 1 Introduction

Let $Y = \{y_1, y_2, \ldots, y_M\}$ be a finite population of which estimating proportion of individuals having sensitive attribute is the prime interest. To procure such data, a respondent may be reluctant to disclose truth or provide incorrect information due to his/her privacy concerns. Therefore, such data collected through conventional (direct) ways are likely to include response-bias. In this connection, Warner (1965) proposed the randomized response method with objective to collect trustworthy data by protecting privacy of the interviewee (respondent). This method, based on a randomizing device, requires interviewee to report 'yes' or 'no' as per statement pointed out by the randomizing device without revealing it to the interviewer. This technique is quite encouraging as respondent cannot be identified i.e., which question he/she has answered. After development of this pioneer work in SRS framework, different researchers have proposed a verity of methods which protect respondent's privacy by different ways and (or) give precise estimate under SRS scheme. For example, see Horvitz et al. (1967), Greenberg et al. (1969), Kuk (1990), Mangat (1994) and reference therein.

McIntyre (1952) advised the RSS scheme as oppose to the conventional SRS scheme for investigation of population characteristics. The RSS method considers ranking of the small sets of units by eyes

*Corresponding author. E-mail: abbasiqau2007@yahoo.com

or using an auxiliary variable before acquiring final sample. Thereafter, Takahasi and Wakimoto (1968) have developed the mathematical setup of RSS procedure. A comprehenvie literature with application of RSS can be seen, among others, in Stokes (1977), Frey (2011), Al-Omari and Haq (2012), Zamanzade and Mohammadi (2016), Zamanzade and Mahdizadeh (2018).

Terpstra and Liudahl (2004) have investigated usual (insensitive) population proportion using RSS scheme and then compared with SRS rival. Following Terpstra and Liudahl (2004), Zamanzade and Mahdizadeh (2017) suggested a new proportion estimator in RSS scheme and claimed that it works better than Terpstra and Liudahl (2004). Later on, Abbasi and Shad (2021a) has pointed out some bottlenecks associated with Zamanzade and Mahdizadeh (2017) estimator and suggested new improved estimators in RSS schemes.

Recently, Abbasi and Shad (2021) have introduced a new sensitive proportion estimator in RSS scheme that beats SRS-based Warner (1965) competitor. In this study, keeping in view different perceptions of sensitive inquiry, we suggest a mixed (partial randomized response) method for acquiring reliable data using RSS scheme, wherein ranking is done via a continuous auxiliary variable. We discuss its properties and compare with SRS competitor. The rest parts of this study are arranged as: A review of existing sensitive proportion estimators is given in Section 2. The proposed estimator, along with its properties, is developed in Section 3. Application to AIDS data is considered in Section 4. Cost analysis is done in Section 5, and final remarks are included in Section 6.

## 2 A review of existing methods

Let $Y_{1j}, Y_{2j}, \ldots, Y_{mj}$ denotes a simple random sample with replacement sampling (SRSWR) of size $m$ in $j$th cycle, for $j = 1, 2, \ldots, r$. To collect true response, each interviewee is provided with a randomizing device, say a spinner, to choose one of the following two assertion, without revealing it to the interviewer:

**(a)** I have the sensitive attribute A

**(b)** I do not have the sensitive attribute A

with, respective, pre-determined selection probabilities $p \neq 0.5$ and $1-p$. Each interviewee uses the said device and reports 'yes' or 'no' as per selected statement and his/her correct status. Then 'yes' response of $i$th $(i = 1, 2, \ldots, m)$ respondent at $j$th cycle can be written as

$$\lambda = Pr(Y_i = 1) = p\pi + (1-p)(1-\pi)$$

Let $m_1$ be the total number of 'yes' responses from the sample size $N = mr$. Warner (1965) established the maximum likelihood (ML) estimate of $\pi$ as given by $\hat{\pi}_w = \{\hat{\lambda} - (1-p)\}/(2p-1)$, $\hat{\lambda} = m_1/N$ and showed that $\hat{\pi}_w$ is unbiased and its variance is given by

$$\text{Var}(\hat{\pi}_w) = \frac{1}{N}\left[\sigma_y^2 + \frac{p(1-p)}{(2p-1)^2}\right]$$

Let $Y$ be a Bernoulli variate and $X$ is a continuous insensitive auxiliary variable having cumulative distribution function (cdf) $F_X(x)$. Let $Y$ given $X = x$ is also Bernoulli i.e., $B(1, g(x))$, where $g(x)$ is a function with range $(0, 1)$. From Abbasi and Shad (2021a), the marginal distribution of $Y$ is

B(1, $\pi$) with parameter $\pi$ = E[$g(X)$], 'E' stands for expected value . In this study we assume that $\pi$ = E[$g(\alpha + \beta X)$], $\alpha, \beta \in \Re$, $g(\cdot)$ is a inverse logit or probit link function, and $X$ follows normal distribution with mean 2 and variance 1 or standard uniform distribution.

To select $m$ units, the RSS scheme that based on an auxiliary variable $X$ can be described as: (i) Identify $m^2$ bivariate items from ($Y$,$X$) and partition them into $m$ sets each of size $m$. (ii) In each set, obtain the exact measurement on $X$, and arrange $Y$ with respect to $X$. (iii) Select $Y$ values corresponding to $i$th ($i = 1, 2, \ldots, m$) ordered unit of $X$ in the $i$th set. The steps (i)-(iii) can be cycled for $r$ times, if desired, to get a final sample of size $N = mr$.

Let $\{(Y_{[i]j}, X_{(i)j}) : i = 1, 2, \ldots, m; j = 1, 2, \ldots, r\}$ be a RSS of size $N$, where $Y_{[i]j}$ denotes $i$th *imperfect* ranked unit in $j$th cycle, and $X_{(i)j}$ serves $i$th *perfect* ranked unit emerged in $j$th cycle. From Terpstra and Liudahl (2004), $Y_{[i]}$ is $B(1, \pi_{[i]})$ with mean $\pi_{[i]} = \mathrm{E}\left[g(X_{(i)})\right]$ and variance $\sigma^2_{y_{[i]}} = \pi_{[i]}(1 - \pi_{[i]})$. Terpstra and Liudahl (2004) introduced insensitive proportion ($\hat{\pi}'_t$) in RSS and is given by

$$\hat{\pi}'_t = \frac{1}{N} \sum_{i=1}^{m} \sum_{j=1}^{r} Y_{[i]j} \tag{2.1}$$

Let the respondents under RSS scheme are directed to select one of the two aforesaid statements (a) and (b) by using Warner (1965) randomizing device. The respondent reports 'yes' ('no') according to the outcomes of the device and his/her actual status. Let $Y_{[i]j} = 1$ if $i$th ranked unit reports 'yes'. Then

$$Pr(Y_{[i]j} = 1) = p\pi_{[i]} + (1 - p)(1 - \pi_{[i]})$$

Let $Y^*_{[i]1}, Y^*_{[i]2}, \ldots, Y^*_{[i]r}$ are independent and identically distributed (i.i.d) Bernoulli randomized responses with parameter $p\pi_{[i]} + (1 - p)(1 - \pi_{[i]})$, then ML estimate of $\pi_{[i]}$ for the given data $Y^*_{[i]j}, j = 1, 2, \ldots r$ is $\hat{\pi}_{[i]} = \{\hat{\lambda}_{[i]} - (1 - p)\}/(2p - 1)$, $i = 1, 2, \ldots, m$, where $\hat{\lambda}_{[i]} = z_i/r$; $z_i = \sum_{j=1}^{r} Y^*_{[i]j}$ is the total number of successes observed under $i$th ranked unit. Now, overall measure of $\pi$, using the relation $\pi = \frac{1}{m} \sum_{i=1}^{m} \pi_{[i]}$, see Abbasi and Shad (2021b), is given by

$$\hat{\pi}_a = \frac{1}{N} \left[ \frac{\sum_{i=1}^{m} \sum_{j=1}^{r} Y^*_{[i]j} - N(1 - p)}{2p - 1} \right] \tag{2.2}$$

and its associated variance is

$$\mathrm{Var}(\hat{\pi}_a) = \frac{1}{N} \left[ \frac{1}{m} \sum_{i=1}^{m} \sigma^2_{y[i]} + \frac{p(1 - p)}{(2p - 1)^2} \right]$$

# 3 The Proposed method

In this section, we propose a partial randomized response model to gather reliable data for estimation of population sensitive proportion under RSS scheme. This technique is applicable in the situation in which some respondents prefer 'direct response' to 'randomized response' for a sensitive inquiry. Suppose, taking into account cost and time, the experimenter decides to collect $k \geq 2$ 'direct responses' using Terpstra and Liudahl (2004) method and the remaining $m - k \geq 2$ units through randomized response technique suggested by Abbasi and Shad. Assuming that $k^2 + (m - k)^2$ identifiable units are available and following the lines of (2.1) and (2.2), we propose a mixed (partial randomized response)

model as given by

$$\hat{\pi}_k = \frac{1}{N}\left[\sum_{i=1}^{k}\sum_{j=1}^{r} Y_{[i]j} + \frac{\sum_{g=k+1}^{m}\sum_{j=1}^{r} Y^*_{[g]j} - N'(1-p)}{2p-1}\right], \tag{3.1}$$

$N' = (N - rk)$. It is obvious that $Y_{[i]j}$ and $Y^*_{[g]j}$ are Bernoulli variates with, respective, parameter $\pi_{[i]}$ and $\lambda_{[g]} = (2p-1)\pi_{[g]} + (1-p)$. It is noteworthy that $\hat{\pi}_a$, $\hat{\pi}'_t$ and $\hat{\pi}_w$ are special cases of $\hat{\pi}_k$. For $k = 0$, $\hat{\pi}_k$ reduces to $\hat{\pi}_a$. Similarly, for $k = m$, $\hat{\pi}_k$ becomes $\hat{\pi}'_t$; when $k = 0$ and $m = 1$, $\hat{\pi}_k$ simplifies to $\hat{\pi}_w$

**Lemma**: The proposed estimator $\hat{\pi}_k$ possesses the following properties:

**(i)** It is an unbiased estimator i.e., E($\hat{\pi}_k$)=$\pi$

**(ii)** It is more precise than $\hat{\pi}_a$ and $\hat{\pi}_w$ i.e., Var($\hat{\pi}_k$) $\leq$ Var($\hat{\pi}_a$) $\leq$ Var($\hat{\pi}_w$)

**Proof**:

(i) From (3.1), we have

$$\begin{aligned}
\mathrm{E}(\hat{\pi}_k) &= \frac{1}{N}\left[\sum_{i=1}^{k}\sum_{j=1}^{r} \mathrm{E}(Y_{[i]j}) + \frac{\sum_{g=k+1}^{m}\sum_{j=1}^{r} \mathrm{E}(Y^*_{[g]j}) - N'(1-p)}{2p-1}\right] \\
&= \frac{1}{N}\left[r\sum_{i=1}^{k}\pi_{[i]} + \frac{r\sum_{g=k+1}^{m}\big((2p-1)\pi_{[g]} + (1-p)\big) - N'(1-p)}{2p-1}\right] \\
&= \frac{1}{m}\left[\sum_{i=1}^{k}\pi_{[i]} + \sum_{g=k+1}^{m}\pi_{[g]}\right] \\
&= \frac{1}{m}\sum_{i=1}^{m}\pi_{[i]} \\
&= \pi
\end{aligned}$$

This completes proof (i).

To prove (ii), again using (3.1), we have

$$\begin{aligned}
\mathrm{Var}(\hat{\pi}_k) &= \frac{1}{N^2}\left[\sum_{i=1}^{k}\sum_{j=1}^{r} \mathrm{Var}(Y_{[i]j}) + \frac{\sum_{g=k+1}^{m}\sum_{j=1}^{r} \mathrm{Var}(Y^*_{[g]j})}{(2p-1)^2}\right] \\
&= \frac{1}{N^2}\left[r\sum_{i=1}^{k}\sigma^2_{y[i]} + \frac{r\sum_{g=k+1}^{m}\lambda_{[g]}(1-\lambda_{[g]})}{(2p-1)^2}\right] \\
&= \frac{1}{mN}\left[\sum_{i=1}^{k}\sigma^2_{y[i]} + \sum_{g=k+1}^{m}\sigma^2_{y[g]} + (m-k)\frac{p(1-p)}{(2p-1)^2}\right] \\
&= \frac{1}{N}\left[\frac{1}{m}\sum_{i=1}^{m}\sigma^2_{y[i]} + (1-f)\frac{p(1-p)}{(2p-1)^2}\right] \\
&= \mathrm{Var}(\hat{\pi}_a) - \frac{f}{N}\frac{p(1-p)}{(2p-1)^2}, \quad f = k/m
\end{aligned} \tag{3.2}$$

Since the term $\frac{f}{N}\frac{p(1-p)}{(2p-1)^2}$ is always greater than or equal to zero, it is easy to observe that Var($\hat{\pi}_k$) $\leq$ Var($\hat{\pi}_a$). Moreover, Abbasi and Shad (2021b) have showed that Var($\hat{\pi}_a$) $\leq$ Var($\hat{\pi}_w$). This completes

proof (ii).
The relative precision (RP) of $\hat{\pi}_k$ with respect to $\hat{\pi}_w$ can be evaluated by the formula

$$\mathrm{RP}(\hat{\pi}_k, \hat{\pi}_w) = \frac{\mathrm{Var}(\hat{\pi}_w)}{\mathrm{Var}(\hat{\pi}_k)} \tag{3.3}$$

Following Abbasi and Shad (2021b), the limiting distribution of the proposed estimator, when $m$ is fixed and $r \to \infty$, is given by

$$\sqrt{N}(\hat{\pi}_k - \pi) \to \mathrm{Normal}\left(0, \frac{1}{m}\sum_{i=1}^{m} \sigma^2_{y[i]} + (1-f)\frac{p(1-p)}{(2p-1)^2}\right). \tag{3.4}$$

Some interesting results can be obtained from (3.4). For $k = 0$, it becomes Abbasi and Shad (2021b) result. Furthermore, for $k = m$ or $p = 0(1)$, it gives Terpstra and Liudahl (2004) result under direct inquiry about the attribute of interest. Moreover, in the situation when both $p$ and $m$ are equal to 1, we get conventional method of direct interaction with the respondent under SRS. Note that these are not frequent occurrences; but can happen. Finally, the variance given in (3.4) can be estimated by substituting $\pi_{[i]}$ by $\hat{\pi}_{[i]} = \sum_{j=1}^{r} Y_{[i]j}/r$. In this way, the estimated variance of $i$th ranked unit becomes independent of $g(\cdot)$. This validates asymptotic inference from the suggested method.

## 3.1 Comparison of proportion estimators

In this section, we examine behavior of RP given in (3.3) under inverse logit link function $g(\alpha + \beta X)$ for different choices of $(\beta_0, \beta_1) \in \{(-10, 7), (-5, 5), (-2, 6)\}$, $p \in [0.1, 0.9]$ and assuming $X$ follows (i) normal distribution with parameters mean=2 and variance =1 i.e, N(2,1) (ii) uniform over the range 0 and 1 i.e., U(0,1). It is pertinent to mention that the RP formula is independent of $r$, hence we take different $m(= 4, 5)$ instead of $r$ to examine the performance of $\hat{\pi}_k$. Furthermore, the magnitude of correlation coefficient between $X$ and $Y$ is also computed under inverse logit link function $g(\alpha + \beta X)$ against each above assumed pair $(\beta_0, \beta_1)$, when $X$ is N(2,1) and U(0,1). The resultant $\rho$ values are close to $\{0.7, 0.6, 0.5\}$ and $\{0.6, 0.4, 0.1\}$. Note that the results are obtained by numerical integration technique, using Mathematica Software.
In Figure 1, we have presented RP results obtained when inverse logit link function is used, and $X$ follows above mentioned distributions and $m = 4$. Similarly, Figure 2 depicts RP results under same setup, except, when $m = 5$.
It can be observed from Figures 1-2 that RP is an increasing function of $m(k)$ and/(or) $\rho$. It is also symmetric about $p = 0.5$. In other words, for given $m$ and $\rho$, one can either choose $p$ or $1-p$ for sensitive statement (a) without compromising RP value. For a fixed $m$, as expected, a maximum(minimum) gain in term of RP is achieved at the largest (smallest) value of $k$. Recall that at minimum $k = 0$, RP curve shows behavior of Abbasi and Shad (2021b) estimator and it falls at the bottom position (showing less precise) as compared to the other cases when $k > 0$. During this study, we have also examined behavior of RP values under inverse probit link function. However, these are not displayed – being almost same as those of inverse logit link function.

# 4 Application

We have first collected a real age data set of 50 AIDS patients from a local government hospital, Rawalpindi, Pakistan. Then each respondent was approached and requested to answer the question-whether he/she has had a sexual relation with any sex-worker or otherwise. The respondent was given option to either respond directly or via Warner (1965) randomizing device with $p = 0.2$ and $1-p = 0.8$ respectively. All interviewees were also assured of their identity will never be disclosed, and made it clear that this information could help in their treatments. They were convinced enough that almost half-25 patients gave consent to disclose true information directly. After converting 'yes' and 'no' responses into binary response, we have computed a true sensitive proportion $\pi = 0.523$. We have also computed mean and variance of $X$ and its correlation with $Y$ as given by $\bar{X} = 35.88$ $\sigma_x^2 = 121.66$ and $\rho_{X,Y} = 0.417$. The main purpose of this survey was to make known of the true proportion, and compare performance of the above discussed estimators.

## 4.1 Estimation of sensitive proportion

The data acquired in this section are, now, used to estimate sensitive proportion under Warner (1965) and proposed model. To draw a sample of size $N = 8$ under partial randomized response model with $m = 4$, the following process is repeated for $r = 2$ times. Assuming $k = 0$, draw $m^2$ units and divide them into $m$ sets of size $m$. The units in different sets are ranked with respect to $X$ and then arranged $Y$ accordingly. The obtained data is displayed in Table 1. The last column of Table 1 presents randomized response data. Similarly, the Table 2 gives layout of the data when $k = 2$. Note that in Tables 1-2, $Y_{[a]bc}$ denotes $a$th imperfect ranked unit at $b$th set in $c$th cycle. However, $b$th set information is omitted, from the final data given the last columns of the Tables 1-2, for simplicity. Using these Tables, we have computed $\hat{\pi}_0 = 0.702$, $\hat{\pi}_2 = 0.550$. To estimate sensitive proportion under Warner (1965) procedure, we drew a sample of size $N = 8$ by SRS method and got $\hat{\pi}_w = 0.723$.

## 4.2 Performance evaluation

In this subsection, we assess relative performance of the proposed estimator with respect to SRS scheme using above-mentioned AIDS data. To this end, a comprehensive simulation study is conducted for $m = 4, 5$, $r = 1$ and $k = 0, 2, 3$, and estimates from $\hat{\pi}_w$ and $\hat{\pi}_k$ are computed for $p = 0.2$. Using these numerical results, we have computed their RP values and shown in Table 3.

The results given in Table 3 are consistent with those of plotted in Figures 1-2. Thus, the partial randomized response is an efficient alternative to the existing $\hat{\pi}_a$ and $\hat{\pi}_w$, and can be used confidently to obtain accurate estimate without increasing sampling cost.

# 5 Cost analysis

In survey sampling, several factors such as cost , time and accuracy or precision are taken into account to choose an appropriate sampling design. Generally, cost is main focus in almost all sampling surveys. However, this important factor was neglected in the previous Sections by assuming that there is no cost associated with the ranking of sampling units.

Following Dell and Clutter (1972), we develop a cost model in RSS to assess performance of the

estimators. Let $c_s$ denotes cost of stratification attached with each measured unit in ranked set sampling. In common practice, this is the cost of drawing $m-1$ units and accomplishing judgment ordering of the $m$ units of a set. Similarly, $c_q$ serves the cost of drawing and quantifying a unit without classification or ranking.

Now, the relative efficiency (RE) is defined as the ratio of the variance of the estimator under SRS and RSS with assumption that the total cost, say $C$, is same for both sampling schemes. Following Dell and Clutter (1972), the RE of $\hat{\pi}_k$ with respect to $\hat{\pi}_w$ is given by

$$\mathrm{RE}(\hat{\pi}_k, \hat{\pi}_w) = \frac{c_q}{c_q + c_s} \frac{\mathrm{Var}(\hat{\pi}_w)}{\mathrm{Var}(\hat{\pi}_k)} \tag{5.1}$$

It is clear from (5.1) that for fixed $c_q$ when $c_s$ varies, the magnitude of RE decreases. Moreover, maximum RE value can be gained when $c_s = 0$. To graphical present behavior of (5.1), we assume different combinations of $(c_q, c_s)$ measured in dollar as $\{(20, 2), (20, 4)\}$. Note that, the values of $\mathrm{Var}(\hat{\pi}_h)$, $h = k, w$ are same as obtained in Subsection 3.1.

In Figures 3-4, we have presented RE value for different combinations of $(c_q, c_s)$ when $m = 4$. Similarly, Figures 5-6 show behaviour of RE when $m = 5$.

As expected, RE rapidly decreases as $c_s$ increases and vice-versa. However, the proposed model still remains superior. Hence, it is appropriate to estimate sensitive proportion using partial randomized response model when there is negligible (minimum) cost involved for ranking of units.

# Conclusion

This study has proposed a partial randomized response model for efficiently estimating sensitive proportion under RSS scheme, wherein ranking is done via a continuous auxiliary variable. Both mathematical and numerical results supported the suggested model relative to the existing SRS-based Warner (1965) model. The graphical representation of the numerical results revealed that values of RP are symmetric about $p = 0.5$, and became larger when $m(k)$ or magnitude of correlation between $X$ and $Y$ increases. Moreover, the limiting distribution of the new model is also discussed and then derived some interesting results from it. Finally, the cost analysis has also been done which also advocated supremacy of the new model. Hence, proposed model is highly recommended for sensitive proportion estimation.

## References

Abbasi, A. M. and M. Y. Shad (2021a). Estimation of population proportion using concomitant based ranked set sampling. *Communications in Statistics-Theory and Methods*, 1–22.

Abbasi, A. M. and M. Y. Shad (2021b). Sensitive proportion in ranked set sampling. *PloS one 16*(8), e0256699.

Al-Omari, A. I. and A. Haq (2012). Improved quality control charts for monitoring the process mean, using double-ranked set sampling methods. *Journal of Applied Statistics 39*(4), 745–763.

Dell, T. and J. Clutter (1972). Ranked set sampling theory with order statistics background. *Biometrics*, 545–555.

Frey, J. (2011). A note on ranked-set sampling using a covariate. *Journal of Statistical Planning and Inference 141*(2), 809–816.

Greenberg, B. G., A.-L. A. Abul-Ela, W. R. Simmons, and D. G. Horvitz (1969). The unrelated question randomized response model: Theoretical framework. *Journal of the American Statistical Association 64*(326), 520–539.

Horvitz, D., B. Shah, and W. Simmons (1967). (1967). the unrelated question randomized response model. In *Proceedings*, pp. 65–72.

Kuk, A. Y. (1990). Asking sensitive questions indirectly. *Biometrika 77*(2), 436–438.

Mangat, N. S. (1994). An improved randomized response strategy. *Journal of the Royal Statistical Society: Series B (Methodological) 56*(1), 93–95.

McIntyre, G. (1952). A method for unbiased selective sampling, using ranked sets. *Australian Journal of Agricultural Research 3*(4), 385–390.

Stokes, S. L. (1977). Ranked set sampling with concomitant variables. *Communications in Statistics-Theory and Methods 6*(12), 1207–1211.

Takahasi, K. and K. Wakimoto (1968). On unbiased estimates of the population mean based on the sample stratified by means of ordering. *Annals of the Institute of Statistical Mathematics 20*(1), 1–31.

Terpstra, J. T. and L. A. Liudahl (2004). Concomitant-based rank set sampling proportion estimates. *Statistics in Medicine 23*(13), 2061–2070.

Warner, S. L. (1965). Randomized response: A survey technique for eliminating evasive answer bias. *Journal of the American Statistical Association 60*(309), 63–69.

Zamanzade, E. and M. Mahdizadeh (2017). A more efficient proportion estimator in ranked set sampling. *Statistics & Probability Letters 129*, 28–33.

Zamanzade, E. and M. Mahdizadeh (2018). Estimating the population proportion in pair ranked set sampling with application to air quality monitoring. *Journal of Applied Statistics 45*(3), 426–437.

Zamanzade, E. and M. Mohammadi (2016). Some modified mean estimators in ranked set sampling using a covariate. *Journal of Statistical Theory and Applications 15*(2), 142–152.

Figure Click here to access/download;Figure;Figures.pdf

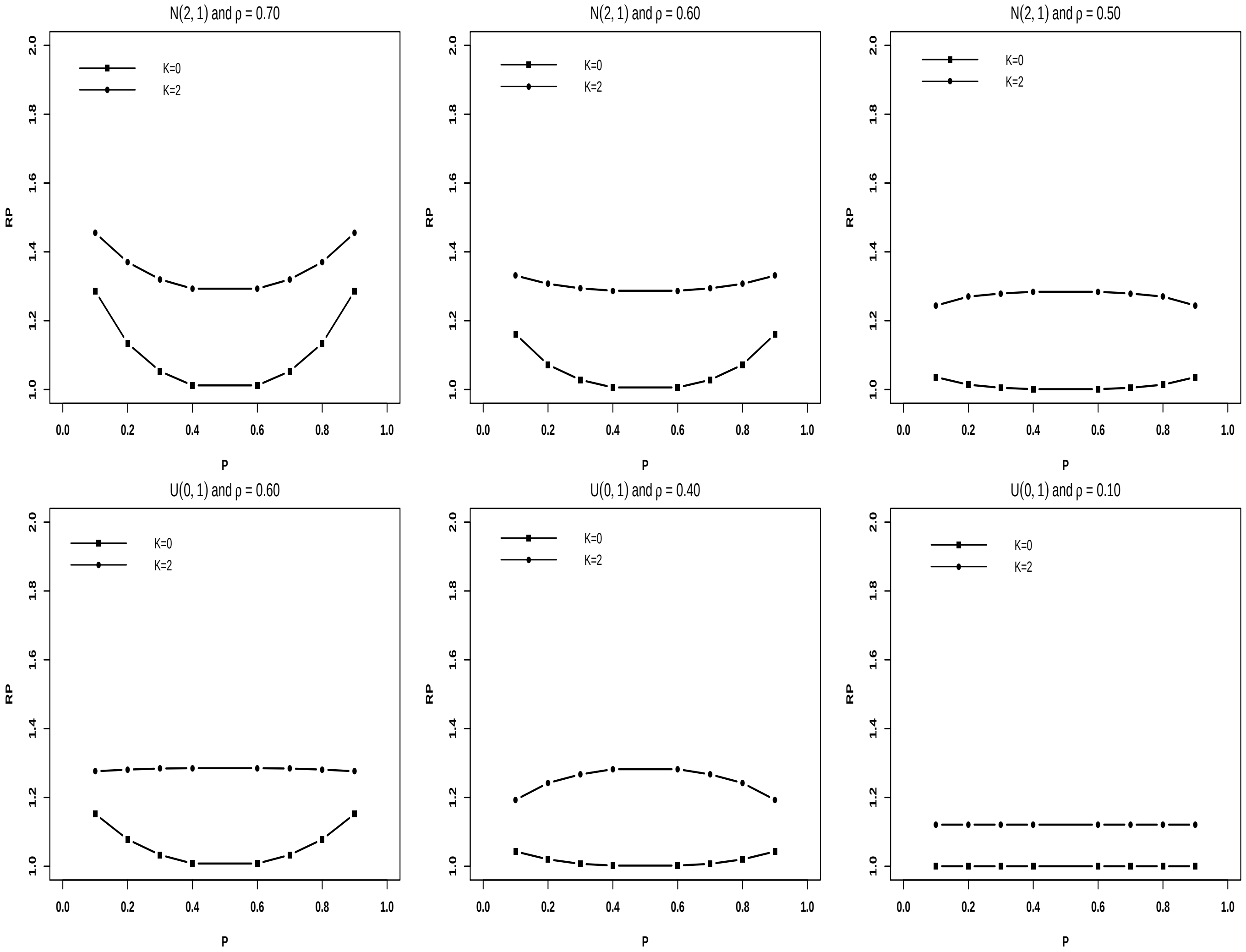


Figure 1: RP of $\hat{\pi}_k$ and $\hat{\pi}_w$ under normal and uniform distributions when $m = 4$

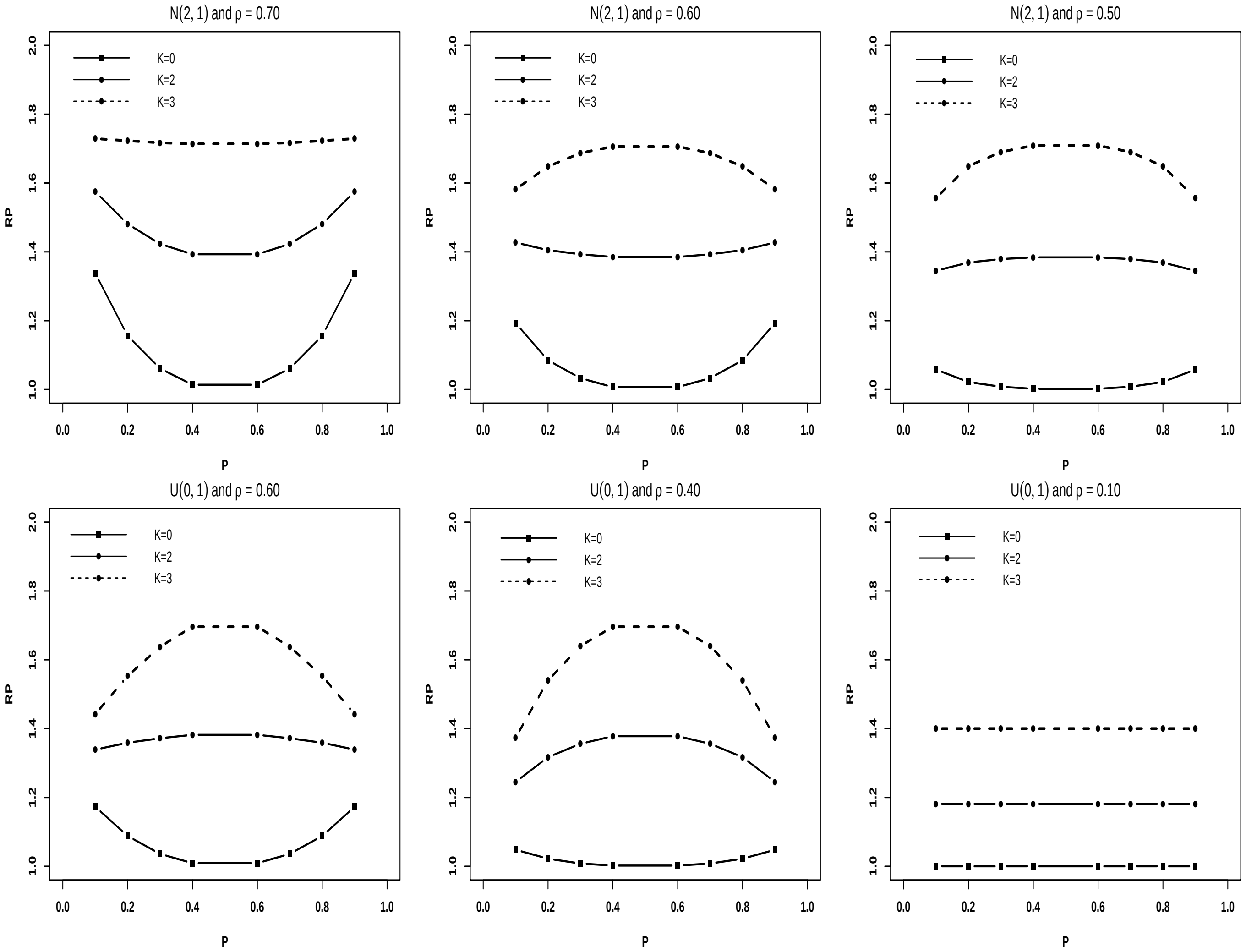


Figure 2: RP of $\hat{\pi}_k$ and $\hat{\pi}_w$ under normal and uniform distributions when $m = 5$

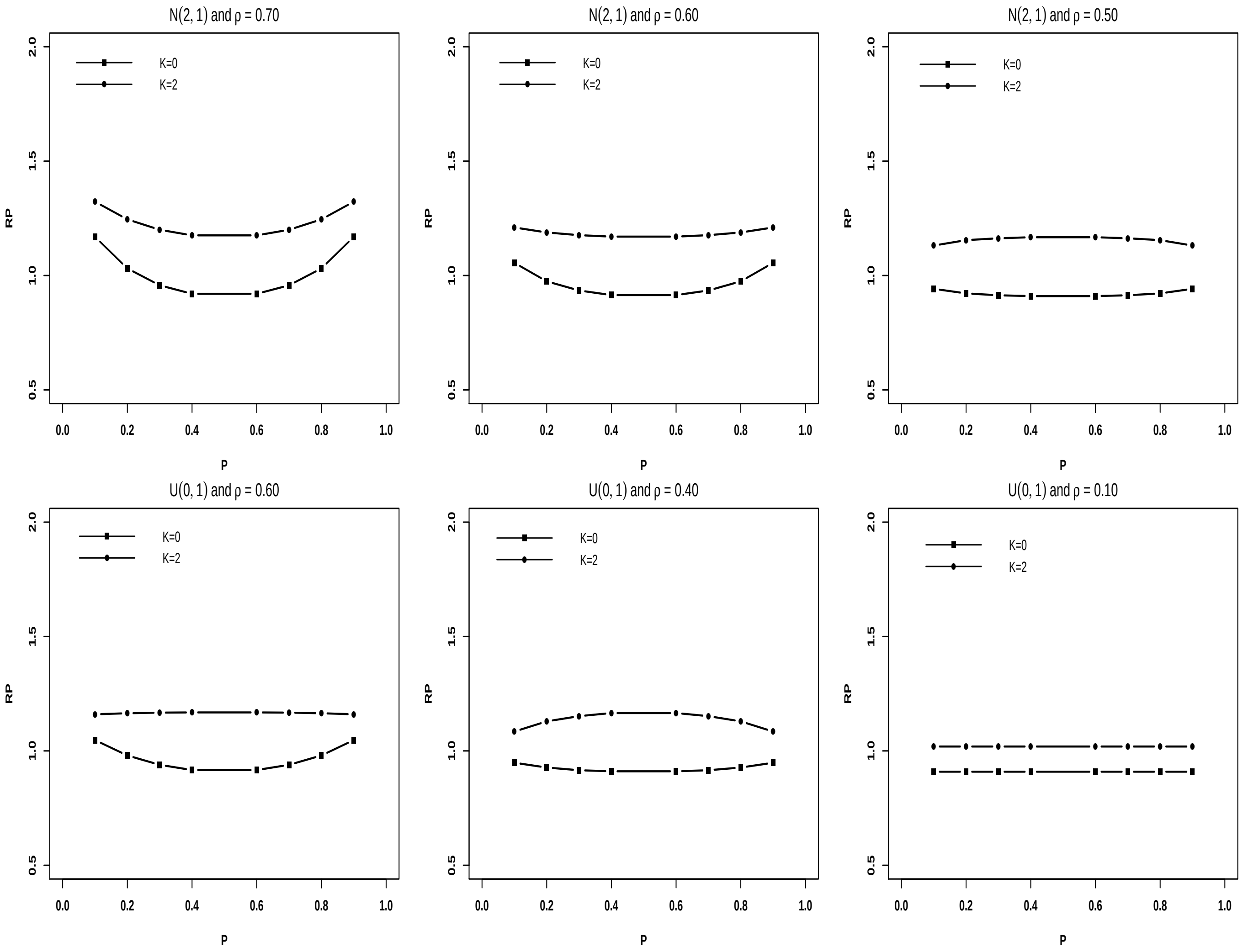


Figure 3: RE of $\hat{\pi}_k$ and $\hat{\pi}_w$ under normal and uniform distribution when $m = 4$, $c_q = \$20$ and $c_s = \$2$

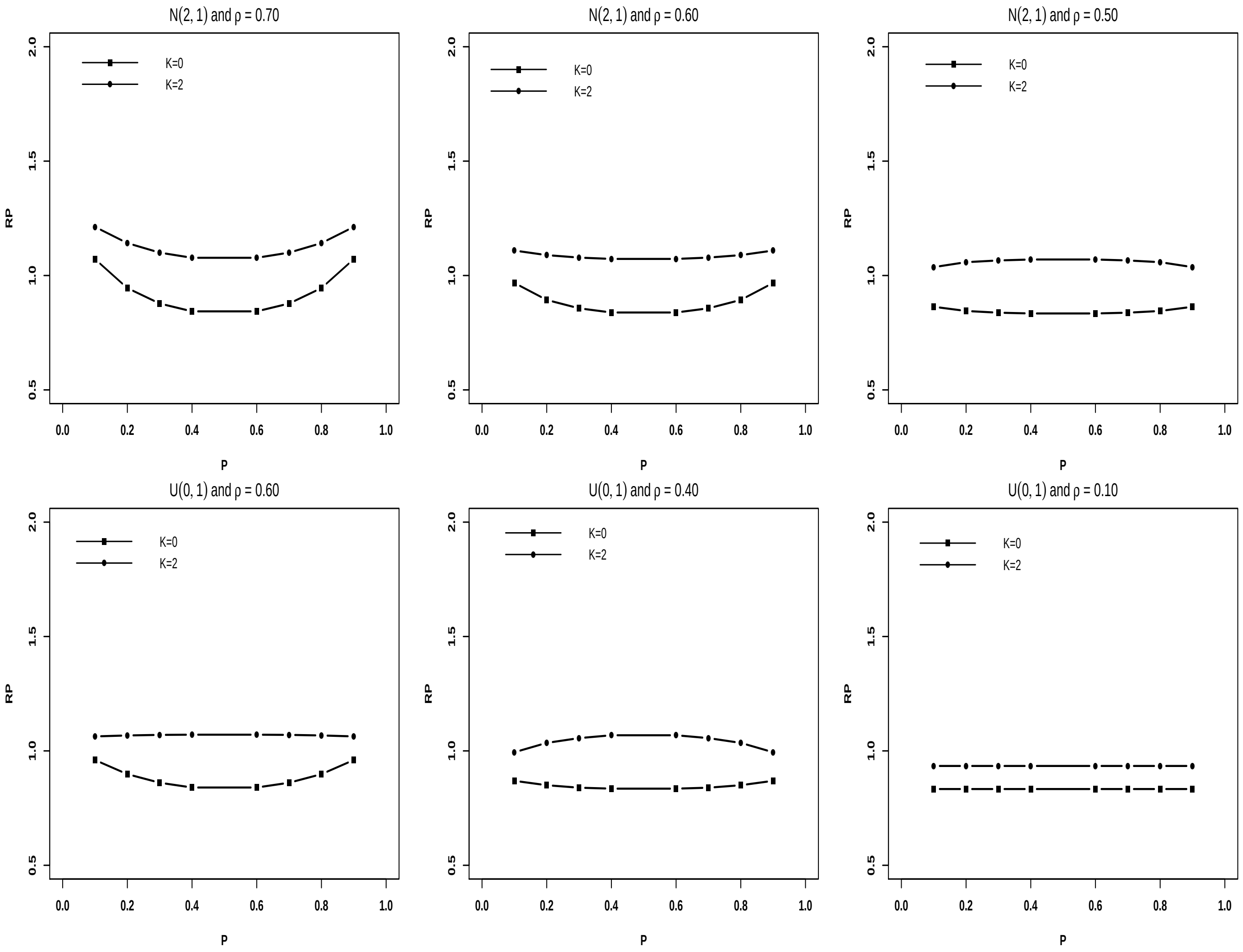


Figure 4: RE of $\hat{\pi}_k$ and $\hat{\pi}_w$ under normal and uniform distribution when $m = 4$, $c_q = \$20$ and $c_s = \$4$

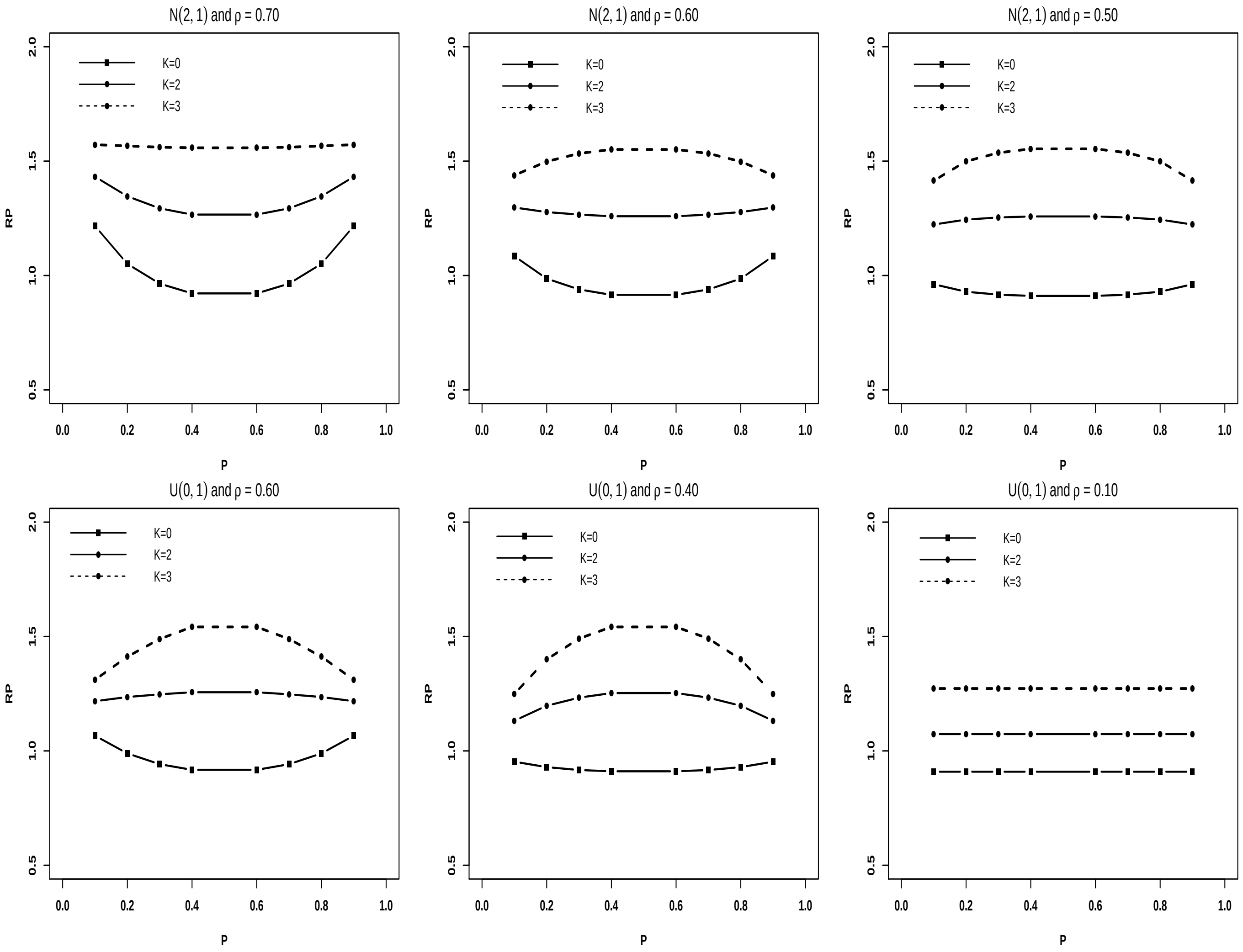


Figure 5: RE of $\hat{\pi}_k$ and $\hat{\pi}_w$ under normal and uniform distribution when $m = 5$, $c_q = \$20$ and $c_s = \$2$

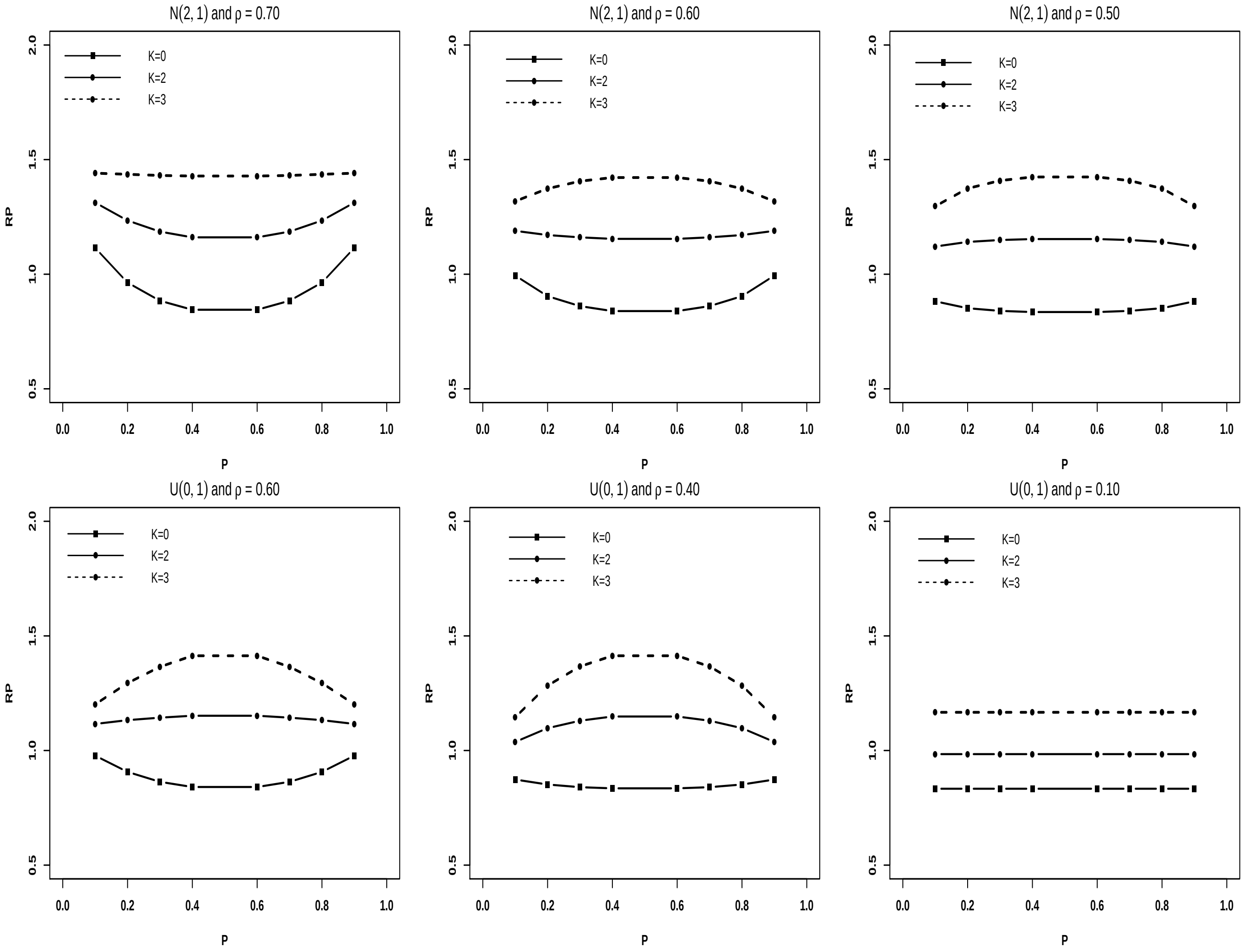


Figure 6: RE of $\hat{\pi}_k$ and $\hat{\pi}_w$ under normal and uniform distribution when $m = 5$, $c_q = \$20$ and $c_s = \$4$
