# Supplementary material for: On partial randomized response model using ranked set sampling
Source: PLoS One. 2022 Nov 29;17(11):e0277497. doi: 10.1371/journal.pone.0277497 (PMC9707803; doi:10.1371/journal.pone.0277497)
Supplement: Attachment — Submitted filename: Response to Reviewers.pdf [file pone.0277497.s019.pdf]

Acknowledgment

The authors are thankful to an Academic Editor and two anonymous reviewers for providing useful comments that led to an improved version of the article.

Response to the Academic Editor

Comment 1: Please ensure that your manuscript meets PLOS ONE's style requirements, including those for file naming.

Answer: As desired, we have amended the manuscript according.

Comment 2: Please include your tables as part of your main manuscript and remove the individual files. Please note that supplementary tables (should remain/ be uploaded) as separate "supporting information" files.

Answer: As desired, we have amended the manuscript according.

Comment 3: Please update your submission to use the PLOS LaTeX template. The template and more information on our requirements for LaTeX submissions can be found at http://journals.plos.org/plosone

Answer: As desired, we have amended the manuscript according.

Comment 4. We note that you have stated that you will provide repository information for your data at acceptance. Should your manuscript be accepted for publication, we will hold it until you provide the relevant accession numbers or DOIs necessary to access your data. If you wish to make changes to your Data Availability statement, please describe these changes in your cover letter and we will update your Data Availability statement to reflect the information you provide.

Answer: We have collected a real data on AIDS and used it to examine the performance of different estimators. Please see S1-S2 Tables in Supporting Information file.

Comment 5. We note you have included a table to which you do not refer in the text of your manuscript. Please ensure that you refer to Table 4-5 in your text; if accepted, production will need this reference to link the reader to the Table.
Answer: It was mistakenly included. We have only three tables (S1-S3 Tables) and mentioned in the manuscript. Please see page 7.

Response to First Reviewer

Comment 1: -page 2: Abbasi and Shad (2021), you must specify the reference year as it is in the bibliography ((2021a) or (2021b)) so that the reader can know to which article you refer.

Answer: As desired, we have amended the references. Please see Page 12.

Comment 2: -page 3: Abbasi and Shad, you must add the year of publication of the article.

Answer: We have amended the manuscript as per PLOS ONE Journal format.

Comment 3: -page 5, 3 The Proposed method: since you explain two models in the review, Warner and Abbasi and Shad model, I think it could be interesting to compare the proposed model with Abbasi and Shad through the relative precision and also add it in the simulation part (3.1 Comparison of proportion estimators), to make the study more complete.

Answer: As desired, we have compared the proposed model with Abbasi and Shad estimator. Please see page 6.

Comment 4: -page 5, 3.1 Comparison of proportion estimators: in the simulation study you use different values of m, rho, k, beta0, beta1, p and various distributions. Why have you chosen these values? Has a previous article served as a reference?

Answer: This is very interesting question. Yes, we have used these values to compare current study with our previous study.

Comment 5:-page 5, 3.1 Comparison of proportion estimators: At the end of the section you indicate that you have used the inverse logit link function, but that you have also examined the behavior of RP with the inverse probit link function. Why have you decided to use the inverse logit link function instead of the inverse probit link function? I think that in addition to indicating with a sentence that the behavior of both is similar, it would be a good idea to include a case in which it can be seen that effectively with both functions the results are similar.

Answer: We (Abbasi and Shad(2021) have conducted a study in the previous year and observed that relative efficiency of the then estimator was almost same under both link functions (Logit and Probit). However, as desired, we have included a case study under probit function in this study (please see page 6)

Comment 6 -page 6, Application: for the analyzes to be reproducible you should include the data and the codes used to obtain the results.

Answer: We have included the data and mentioned the procedure for obtaining the results. Please see page 6.

Comment 7-page 6, 4.1 Estimation of sensitive proportion and 4.2 Performance evaluation: you refer to tables 1, 2, and 3, but these tables are not included in the article, you must include them in the text or at the end as an annex.

Answer: We have included in these tables in the manuscript. Please see S1-S3 Tables in Supporting Information File.

Comment 8 -page 5, 4.2 Performance evaluation: please confirm if I have understood this point correctly. The real study is carried out using the direct technique and using the Warner model and is not carried out with the method proposed by you. This is the reason why you carry out the

simulation study here, to verify what would have happened with your proposed model.

Answer: Yes, real data is carried out by direct and Warner model (randomized response) so that we consider an obtained sensitive proportion as "true proportion" and then compare the performance of all considered estimators by taking sample from the acquired data.

Comment 9: -page 5, 4.2 Performance evaluation: since you explain two models in the review, Warner and Abbassi and Shad model, I think it could be interesting to compare the proposed model with Abbassi and Shad through the relative precision in the simulation part.

Answer: It is a very good suggestion, and we have compared our proposed model with Abbasi and Shad (2021) estimator. Please see Page 7.

Comment 10 -page 5, 4.2 Performance evaluation: in the simulation study you use different values of m, r, k and p. Why have you chosen these values? Has a previous article served as a reference?

Answer: Yes, we have used these values to compare the results of both studies.

comment 11 -page 5, 4.2 Performance evaluation: you write the following sentence at the end of the section and can be used confidently to obtain accurate estimate without increasing sampling cost. I think that in this section you should not refer to the sampling cost, since you are not taking it into account at any time, in any case you could talk about the sampling variance or precision.

Answer: We have omitted the word "cost". please see page 7.

Comment 12: -page 7, 5 Cost analysis: since the cost analysis has a theoretical part and a simulation part, I would explain it before section 4 Application.

Answer: As both theoretical and numerical results of cost analysis are done in the same section 5. Moreover, this Section is a bit different from other Sections. Therefore, we put it here after evaluating performance of different estimators.

Comment 13:-page 7, 5 Cost analysis: since you explain two models in the review, Warner and Abbassi and Shad model, I think it could be interesting to compare the proposed model with Abbassi and Shad through the relative efficiency and also add it in the simulation part to make the study more complete.

Answer: It is very good suggestion, and we have compared the proposed estimator with Abbasi and Shad estimator. Please see Page 8.

Comment 14:-page 7, 5 Cost analysis: in the simulation study you use different values of cq and cs. Why have you chosen these values? Has a previous article served as a reference?

Answer: Yes, we have used these value to compare the results with our previous article.

Comment 15: -page 7, Conclusion: the conclusions are brief. They should give more emphasis to the results obtained so that the readers use the proposed method.

Answer: We have expanded the conclusion of the article.

Comment 16 -page 8, References: Horvitz, D., B. Shah, and W. Simmons (1967). (1967), the year of publication is duplicated. The title of the article should be written in capital letters.

Answer: After transforming the article into PLOS ONE's journal format, this issue stands resolved.

Response to Second Reviewer

Comment 1: Some references are not listed correctly, for instance page 2 "Abbasi and Shad (2021)" a or b ??.

Answer: We have made correction. Please see page 10.

Comment 2: Check grammatical mistake.

Comment 2: We have thoroughly read the article.

Comment 3: 3. Data are not listed and missing!

Answer: Data are included. Please see page 6 and S1-S2 Tables.

Comment 4: Some formulas are not italic.

Answer: Corrected. Please see page 3.

Comment 5: I suggest to add more explanation about RSS since it is a core method in this article.

Answer: It is very good suggestion, and we have included more details about RSS method. Please see page 2
